# AI-based model for automatic identification of multiple sclerosis based on enhanced sea-horse optimizer and MRI scans

**DOI:** 10.1038/s41598-024-61876-9

**Published:** 2024-05-27

**Authors:** Mohamed G. Khattap, Mohamed Abd Elaziz, Hend Galal Eldeen Mohamed Ali Hassan, Ahmed Elgarayhi, Mohammed Sallah

**Affiliations:** 1 Applied Mathematical Physics Research Group, Physics Department, Faculty of Science, Mansoura University, Mansoura, 35516 Egypt; 2 Technology of Radiology and Medical Imaging Program, Faculty of Applied Health Sciences Technology, Galala University, Suez, 435611 Egypt; 3 Faculty of Computer Science and Engineering, Galala University, Suez, 435611 Egypt; 4 Department of Electrical and Computer Engineering, Lebanese American University, Byblos, 13-5053 Lebanon; 5 Diagnostic and Interventional Radiology Department, Faculty of Medicine, Ain Shams University, Cairo, 11591 Egypt; 6 Department of Physics, College of Sciences, University of Bisha, P.O. Box 344, Bisha, 61922 Saudi Arabia

**Keywords:** Multiple sclerosis (MS), Magnetic resonance imaging (MRI), AI-based diagnosis, Feature selection, Sea-horse optimizer (SHO), Multiple sclerosis, Computer science

## Abstract

This study aims to develop an AI-enhanced methodology for the expedited and accurate diagnosis of Multiple Sclerosis (MS), a chronic disease affecting the central nervous system leading to progressive impairment. Traditional diagnostic methods are slow and require substantial expertise, underscoring the need for innovative solutions. Our approach involves two phases: initially, extracting features from brain MRI images using first-order histograms, the gray level co-occurrence matrix, and local binary patterns. A unique feature selection technique combining the Sine Cosine Algorithm with the Sea-horse Optimizer is then employed to identify the most significant features. Utilizing the eHealth lab dataset, which includes images from 38 MS patients (mean age 34.1 ± 10.5 years; 17 males, 21 females) and matched healthy controls, our model achieved a remarkable 97.97% detection accuracy using the k-nearest neighbors classifier. Further validation on a larger dataset containing 262 MS cases (199 females, 63 males; mean age 31.26 ± 10.34 years) and 163 healthy individuals (109 females, 54 males; mean age 32.35 ± 10.30 years) demonstrated a 92.94% accuracy for FLAIR images and 91.25% for T2-weighted images with the Random Forest classifier, outperforming existing MS detection methods. These results highlight the potential of the proposed technique as a clinical decision-making tool for the early identification and management of MS.

## Introduction

Multiple sclerosis (MS) is a chronic autoimmune condition affecting the central nervous system (CNS), leading to considerable neurologic impairment and demyelination among young adults^1^. Although ten percent of patients with MS live for more than 20 years with the disease, half may require walking assistance within 15 years^2^. It is important to note that rapid mortality within months of diagnosis is uncommon and typically results from comorbid conditions rather than MS itself, highlighting the critical need for comprehensive care and management^3^. The global incidence of Multiple Sclerosis is estimated to affect approximately 2.8 million individuals, with a prevalence rate of 35.9 per 100,000 individuals^4^. The symptoms of MS vary from blurred vision to severe muscle weakness and cognitive impairment, depending on the part of the CNS affected^5^. This is caused by the deterioration of the myelin sheath, a fatty material that envelops neural fibers called axons^6^. As there is no known cure for MS, disease management is the only available treatment that should be tailored to each patient’s individual needs^7^.

Currently, magnetic resonance imaging (MRI) findings, laboratory results, and clinical symptoms are utilized to diagnose and evaluate the progression of MS^2^. In conventional T2-weighted MRI sequences, MS lesions are commonly characterized by increased signal intensity, presenting as bright or hyperintense regions, which serve as indicators of inflammation and demyelination. According to the 2017 McDonald criteria^8^, a diagnosis of MS requires evidence of both dissemination in space (DIS) and dissemination in time (DIT) within the CNS. To establish DIS, MRI must reveal one or more T2-hyperintense lesions characteristic of MS in at least two out of four specific CNS areas: periventricular, cortical or juxtacortical, infratentorial regions, and the spinal cord. DIT can be confirmed either by the concurrent presence of gadolinium-enhancing and non-enhancing lesions at any point or by identifying a new T2-hyperintense or gadolinium-enhancing lesion on a subsequent MRI compared to a baseline scan, irrespective of the timing of this initial MRI. Given the enormous economic effect and lower quality of life in MS patients, innovative methods that may deliver a higher diagnostic rate in MS monitoring and earlier treatment initiation are needed. The MS diagnostic process using MRI brain scans is both time-consuming and prone to human error, mainly due to challenges in distinguishing MS from its mimics such as small vessel disease, acute disseminated encephalomyelitis (ADEM), neuromyelitis optica (NMO), and others based on MRI findings, which can lead to misdiagnosis and delays in appropriate management^9^. A recent study across two academic MS referral centers found a misdiagnosis rate of 17-19%, with the most common correct diagnoses being migraine (16%), radiologically isolated syndrome (9%), spondylopathy (7%), and neuropathy (7%). These misdiagnoses contributed to approximately 110 patient-years of unnecessary MS disease-modifying therapies (DMT), underlining the significant patient morbidity and healthcare costs associated with misdiagnosis^10^.

In the past few years, it has become clear that Artificial Intelligence (AI) can help identify abnormalities in medical imaging investigations^1,11,12^. The power of AI to process huge amounts of data is so much greater than that of humans that it has been put to use in the field of MS diagnosis and progression monitoring^13–16^. The great performance of Machine Learning (ML) in this discipline, as well as the improved quality of the findings, opened the door for higher interaction of such technologies. Among the array of techniques employed, metaheuristic algorithms (MAs) have garnered attention for their ability to solve complex optimization problems, including feature selection (FS) in medical imaging analysis^17^. In the realm of MAs, a diverse spectrum of algorithms has been explored for various applications, ranging from engineering optimization to medical image analysis. Notably, algorithms such as the Liver cancer algorithm (LCA)^18^, slime mould algorithm (SMA)^19^, moth search algorithm (MSA)^20^, hunger games search (HGS)^21^, Runge Kutta method (RUN)^22^, colony predation algorithm (CPA)^23^, weighted mean of vectors (INFO)^24^, Harris hawks optimization (HHO)^25^, and rime optimization algorithm (RIME)^26^ have demonstrated significant potential. Each of these algorithms brings unique mechanisms and strategies to the optimization process, inspired by natural phenomena and behaviors. Their applications have led to notable improvements in solving intricate problems, with several studies highlighting enhancements to these algorithms for increased efficiency and accuracy.

This paper aims to develop an AI model using an improved sea-horse optimizer (SHO) algorithm^27^ to differentiate between MS and healthy MRI-scanned brains, thereby supporting radiologists in reaching an initial diagnosis based on a given MRI scan. Furthermore, this could expedite the initiation of treatment with DMT, potentially slowing the progression of the disease^28^. To assess the efficacy of this model and its potential application in MS diagnosis, the primary goal of the modified SHO is to reduce the dimensionality of the features derived from the MS scans. In fact, the SHO algorithm^27^ is motivated by the behavior of seahorses in the sea, including prey hunting, movement, and reproduction, and has found applications in various domains. In engineering optimization, it has demonstrated its utility in addressing complex problems, such as parameter tuning in mechanical systems, optimal structural design, and optimization of control systems^29,30^. Additionally, SHO has been applied to power system optimization, addressing challenges such as optimal power flow, economic dispatch, and unit commitment. By leveraging the algorithm’s capabilities, optimal or nearly optimal controller parameter solutions for complex power system operational issues can be obtained^31–33^. However, the performance of SHO still needs more improvements, especially its exploitation ability. This motivated us to present a modified version of SHO using the Sine Cosine Algorithm (SCA)^34^.

Since SCA has established its quality in different applications. For example, one of the key uses of the SCA algorithm is in engineering and design optimization^35^, where it is employed to find optimal solutions for problems such as economic load dispatch and radial distribution networks. It also excels in optimal load frequency control, Phasor Measurement Unit placement, hybrid power generation system design, unit commitment, and bend photonic crystal waveguide design. In addition, SCA has the ability to tune controller parameters and enhance the lifetime of wireless sensor networks. In classification, SCA is effective in reducing the feature size, as evaluated based on 18 datasets from the UCI ML repository^36^. In image processing, it excels in image binarization and curve fitting.

In general, the developed AI-based MS model starts by extracting the features from the collected MS images. This was achieved using the first-order histogram-based features, gray level co-occurrence matrix (GLCM), and local binary pattern (LBP) features. Then, to select the relevant features, a modified version of the SHO algorithm based on SCA was employed. This hybrid approach utilizes the strengths of both algorithms to effectively identify and choose the most pertinent features for the model.

The main contributions of this study are summarized as:

- Propose an AI-based model to aid radiologists in the early diagnosis of MS, thereby reducing the misdiagnosis with MS mimics.
- Present a novel enhanced feature selection method based on improving the performance of SHO using SCA to determine the optimal feature selection strategy for multiple sclerosis disease detection.
- Apply the developed MS detection model to the open-access eHealth lab dataset as well as a real-world brain MRI dataset for MS diagnosis. In addition, comparing it with the state-of-the-art methods.

## Related work

### The literature of ML for MS detection

Numerous investigations have been conducted utilizing MRI-based ML techniques. To discriminate between individuals with MS and healthy controls (HCs) in MRI, Zhang et al.^37^ developed an ML system using the online eHealth lab dataset, which comprises only 38 MS patients. They employed the SWE method to extract relevant features. Their approach compared decision tree (DT), k-nearest neighbors (kNN), and support vector machine (SVM) classifiers, ultimately identifying kNN as the most effective due to its high specificity, precision, and accuracy in handling low-dimensional feature spaces. Outperforming four state-of-the-art methods, the study highlights the potential of SWE combined with kNN in MS detection. However, the reliance on a relatively small, publicly available dataset from the University of Cyprus limits the study’s scalability and applicability to broader, more heterogeneous populations, underscoring the need for further research into the generalizability and optimization of detection techniques.

Wang and his colleagues^38^ endeavored to develop an innovative method for early-stage MS diagnosis. They utilized 676 MRI images from 38 patients with plaques from the eHealth lab dataset and 880 MRI images from 34 healthy individuals. Using a biorthogonal wavelet transform (BWT), principal component analysis with an RBF kernel (RKPCA), and logistic regression (LR), they introduced a unique classifier approach. PCA was implemented for dimensionality reduction, and KPCA was employed to manage non-linear data, following the feature extraction using Discrete Wavelet Transform (DWT). The model underwent training with binary LR through cross-validation, achieving high sensitivity, specificity, and accuracy. This method is distinguished by its integration of RKPCA for effective dimensionality reduction and its comparative advantage over five contemporary approaches, demonstrating its efficacy in distinguishing MS-affected MRI slices. Despite its methodological innovation and advantages over existing techniques, the study faces limitations, including a reliance on a small public dataset and the exclusive use of T2-weighted MRI sequences. This suggests a need for validation across larger datasets and a broader range of MRI sequences to improve diagnostic applicability and robustness.

Deshpande et al.^39^ utilized MRI data from 13 MS patients to develop a supervised method for automatically classifying MS lesions, employing sparse representations and dictionary learning. The study revealed that increasing the specificity of dictionaries for each anatomical structure in the brain could enhance classification accuracy. This improvement is attributed to the unique intensity patterns these structures present in multi-channel MR images. Additionally, tailoring the dictionary sizes to the complexity of the data can further boost classification results, marking notable progress in the field. Nevertheless, the small size of the dataset may limit the generalizability of the findings. While adaptive dictionary sizes have the potential to improve classification precision by optimizing data representation, concerns about possible overfitting and the computational demands of the approach, particularly with larger datasets, arise. Despite these challenges, Deshpande et al.’s method, which showed increased sensitivity, is promising for enhancing the automated classification of MS lesions. The study suggests that further validation with a larger patient cohort and exploration into computational efficiencies are essential for broader clinical adoption.

In a study by Jain et al.^40^, 18 gray-level textural features derived from MRI data were utilized to compare various ML classifiers. Both unsupervised methods, such as k-means clustering and Gaussian mixture models, and supervised methods, including SVM and KNN, were evaluated across a spectrum of parameters. The dataset consisted of 110 normal MRI images and 82 MS scans. The authors concluded that supervised ML approaches outperformed ML-based clustering techniques in distinguishing between healthy individuals and MS patients, achieving a peak accuracy of 96.55%. However, the study’s reliance on a relatively small dataset and a limited evaluation of unsupervised methods, along with its computational limitations, might restrict the applicability of the findings and overlook the potential of more advanced, computationally demanding models such as DL. Moreover, the exclusive focus on specific textural features raises questions about the impact of alternative or additional image features on diagnostic accuracy. Despite these limitations, Jain et al.’s research significantly contributes to the discussion on the application of ML in detecting neurological diseases, suggesting promising directions for future research, including exploring different feature extraction techniques and integrating convolutional neural networks (CNNs).

Ensemble learning, a classification method, was recently utilized to diagnose MS^41^, achieving a notable accuracy of 94.91%. This research employed 18 unique GLCM features for feature extraction and utilized DT-based ensemble learning for classification. To classify MR images of healthy and unhealthy brains, three boosting approaches were employed: AdaBoost, LogitBoost, and LPBoost. The study’s methodology, from preprocessing to classification across a dataset of 293 images, is distinguished by its comprehensive approach and high performance metrics, demonstrating its effectiveness over traditional neural network and wavelet transform methods. However, the study’s limited comparative analysis with other contemporary techniques and its somewhat vague direction for future work leveraging CNNs for GLCM feature analysis indicate areas for improvement. This holistic view acknowledges the work’s significant contributions to MS diagnosis while recognizing areas where further comparative analyses and technical advancements could enhance understanding and application.

Aoki et al.^42^ developed a model to differentiate between relapsing-remitting MS (RRMS), progressive MS (PMS), and HCs based on MS brain atrophy patterns. The dataset comprised brain volumes of 55 segments obtained from 72 MS patients and 21 healthy controls from Tohoku Medical and Pharmaceutical University Hospital, Japan. Before analysis with supervised machine learning classifiers (Bayesian regularized neural networks (BRNN) and SVM), the data underwent several preprocessing steps, including automatic segmentation, logarithmic conversion, and normalization. The BRNN classifier achieved a sensitivity of 77.8% and a specificity of 95.2%, showcasing its potential as a predictive marker in MS healthcare. While the study highlights the model’s clinical relevance by correlating MS prediction rates with the Expanded Disability Status Scale (EDSS) and providing a quantitative model that links brain atrophy to disease severity, it also acknowledges limitations such as potentially reduced applicability across different racial groups due to the homogeneous Japanese sample and a relatively small sample size. Furthermore, the exclusion of patients with NMO, which is more prevalent in Asian populations, might limit the model’s broader diagnostic utility.

In a recent article by Macin et al.^43^, a novel handcrafted feature extraction strategy was employed to develop a computationally efficient ML model for diagnosing MS. The study utilized a publicly available dataset consisting of brain MRI FLAIR images from 72 MS patients and 59 individuals without the condition. These images were categorized into axial, sagittal, and a combination of both types and were collected in 2021 at Ozal University Medical Faculty. The Exemplar Multiple Parameters Local Phase Quantization (ExMPLPQ), a patch-based model of fixed size, was used for feature extraction, while iterative neighborhood component analysis (INCA) was applied for feature reduction. KNN algorithm was subsequently trained to distinguish between MS patients and HCs, achieving an impressive accuracy of over 97%, thereby surpassing 19 established deep learning models. This was accomplished by applying the ExMPLPQ-based KNN model with 10-fold cross-validation to axial images. However, the study’s applicability to a broader patient population may be limited due to its reliance on a dataset collected from a single institution over one year and specific inclusion criteria, such as excluding patients with fewer than nine MS lesions or those with poor MRI image quality.

### The literature of application of MAs techniques

In their study, Han and Hou^44^ illustrated how wavelet entropy combined with a feedforward neural network (FNN) trained via an adaptive genetic algorithm (AGA) for diagnosing MS. Their methodology involved processing MRI images from both the eHealth Lab dataset and 26 healthy individuals through histogram normalization and utilizing a wavelet decomposition level of 3. Their findings not only confirmed the efficiency of this approach but also highlighted the superiority of AGA over the traditional genetic algorithm (GA).

Wang et al.^16^ demonstrated that extracting fractional Fourier entropy maps from MS images, acquired from the eHealth Lab dataset, and feeding them into the modified Jaya algorithm to train the Multilayer Perceptron (MLP) classifier further resulted in excellent accuracy. Zhou and Shen^45^ have introduced a novel approach for detecting MS lesions in MRI images. Their approach makes use of BBO training methods and GLCM feature extraction. They utilized images from two separate sets: 38 MS patients and 26 healthy individuals. The BBO technique was utilized for training the multilayered FNN classifier. The outcomes supported high sensitivity, specificity, and accuracy. Han et al.^46^ introduced an approach to MS recognition which integrates Hu moment invariants (HMI) as an image analysis technique for feature extraction, artificial neural networks (ANN) as a classifier, and a well-known metaheuristic algorithm named particle swarm optimization (PSO) to augment the accuracy and effectiveness of MS detection and classification.

Numerous studies have embarked on exploring the efficacy of FS techniques in medical image analysis by leveraging the capabilities of MAs, aiming to enhance the precision and efficiency of various computational models across diverse domains. For instance, Rezaee et al.^47^ employed Fractal and Pseudo-Zernike moments techniques to extract features and generate a feature vector from MRI scans for the detection of MS. They used the Differential Evolution algorithm for FS, thereby reducing the inputs for the Extreme Learning Machine (ELM) classifier, which was subsequently fine-tuned using the Shuffled Frog-Leaping algorithm to enhance its wavelet kernel parameters. Testing on brain images from 61 healthy individuals and 64 MS patients, collected from Vasei Hospital in Iran, demonstrated remarkable accuracy through 5-fold cross-validation.

Houssein and Sayed^48^ developed an enhanced version of the weighted mean of vectors (mINFO) algorithm, integrating Opposition-Based Learning (OBL) and Dynamic Candidate Solution (DCS) strategies to optimize FS for Chronic Kidney Disease (CKD) classification. Utilizing the kNN classifier, their approach was rigorously evaluated on complex datasets, including the CEC’22 test suite and UCI’s CKD datasets. The mINFO algorithm demonstrated superior performance in comparison to several established metaheuristic algorithms, achieving an impressive classification accuracy of 93.17% on CKD datasets. This study represents a significant contribution to the application of MAs in medical data classification, showcasing the potential of mINFO for enhancing diagnostic accuracy in healthcare.

Abd Elaziz et al.^49^ introduced an Improved Artificial Rabbits Optimizer (IARO) integrating Gaussian mutation and crossover operator for effective FS in skin cancer prediction. Their approach employs the MobileNetV3 architecture for feature extraction from dermoscopic images across PH2, ISIC-2016, and HAM10000 datasets, achieving notable accuracies of 87.17% on ISIC-2016, 96.79% on PH2, and 88.71% on HAM10000. This study highlights IARO’s superiority in optimizing FS, enhancing the predictive accuracy of skin cancer detection algorithms, and offering a significant contribution to medical image analysis, especially in automating the skin cancer detection process.

El-Kenawy et al.^50^ proposed a novel approach for COVID-19 classification using CT images, incorporating Guided Whale Optimization Algorithm (Guided WOA) for FS and a novel voting classifier for classification. Their method begins with feature extraction from CT scans using AlexNet, followed by FS via a modified WOA enhanced with Stochastic Fractal Search (SFS), aimed at selecting valuable features. The selected features were then balanced, and a voting classifier, leveraging PSO and Guided WOA techniques, was introduced to aggregate predictions from SVM, KNN, and DT classifiers. Tested on two datasets, their framework demonstrated superior performance, with an AUC of 0.995, outperforming traditional algorithms. This study highlights the potential of combining advanced FS with ensemble learning techniques for improving diagnostic accuracy in medical imaging.

Dey et al.^51^ proposed a hybrid meta-heuristic FS model, Manta Ray Foraging based Golden Ratio Optimizer (MRFGRO), for efficient COVID-19 detection from CT images. Leveraging fine-tuned CNNs, specifically GoogleNet and ResNet18, for deep feature extraction, they developed MRFGRO to select the most significant feature subset for classification. Implemented on three publicly available datasets-COVID-CT, SARS-CoV-2, and MOSMED-the model demonstrated state-of-the-art accuracies of 99.15%, 99.42%, and 95.57%, respectively. This approach not only validates the efficacy of hybrid meta-heuristic algorithms in medical image analysis but also emphasizes their potential in enhancing diagnostic accuracies for diseases like COVID-19.

## Background

### Feature extraction

In the domains of image analysis and computer vision, feature extraction stands as a crucial first step, entailing the selection of relevant patterns or features from images for further processing tasks like object recognition or classification. These patterns form the basis for training ML algorithms to identify similar features in new images. Techniques for feature extraction vary, encompassing traditional methods like edge and corner detection^52^, as well as texture analysis methods such as statistical analysis, Fourier analysis, wavelet analysis, and local binary patterns (LBP)^53^. Specifically, this research focuses on applying these methodologies to brain MRI images, aiming to extract distinct features including first-order histogram-based features, Gray Level Co-occurrence Matrix (GLCM), and LBP features, which are briefly covered here.

#### First-order histogram-based features

Mean, variance, standard deviation, skewness, kurtosis, and entropy are useful first-order statistical features. In order to compute these features, the following formulae are presented^54^.

**The Mean** represents the image’s overall intensity level and is calculated by adding all the image’s pixel intensities and then dividing by the number of pixels in the image. It is given by:

1 $$\begin{aligned} m=\frac{1}{N} \sum _i \sum _j I(i, j) \end{aligned}$$

**The Variance** is a histogram width metric that quantifies the variability of the intensity variations relative to the mean and is given by:

2 $$\begin{aligned} {\text {var}}=\sum _i \sum _j(i-m)^2 I(i, j) \end{aligned}$$

**The standard deviation** refers to a statistical measure that describes the amount of variation in pixel intensities within a certain neighborhood or region of interest in an image. A higher value indicates greater diversity in the values and high contrast of the edges of an image. The standard deviation is defined by:

3 $$\begin{aligned} {\text {Std}}=\sqrt{\frac{\sum _i \sum _j(I(i, j)-m)^2}{N}} \end{aligned}$$

**Skewness** quantifies the asymmetry or lack of symmetry in the distribution of pixel values around the mean and is denoted as:

4 $$\begin{aligned} \text{ Skewness } =\frac{1}{N} \frac{\sum _i \sum _j(I(i, j)-m)^3}{{\text {std}}^3} \end{aligned}$$

**Kurtosis** is a measure of the degree of flatness of a distribution compared to a normal distribution and is represented by:

5 $$\begin{aligned} \text{ Kurtosis } =\frac{1}{N} \frac{\sum _i \sum _j(I(i, j)-m)^4}{{\text {std}}^4} \end{aligned}$$

**Entropy** is calculated to measure the randomness amount of intensity values in an image. Entropy is defined as:

6 $$\begin{aligned} \text{ Entropy } =-\sum _{k=0}^{L-1} h(k) \ln [h(k)] \end{aligned}$$

where *N* is the total number of pixels in the image. *I*(*i*, *j*) represents the pixel intensity.

#### Gray level co-occurrence matrix features (GLCM)

First-order statistical features are helpful in exposing details about the distribution of pixel intensities in an image. Nonetheless, they don’t offer any information about how the various pixels are positioned in relation to one another. Consequently, these features cannot determine whether low-value pixels are located next to one another or intermingled with high-value pixels^55^. The GLCM quantifies the spatial relationship between pairs of pixels in an image by computing the frequency of occurrence of different pixel intensity combinations at a specific distance (d) and orientation ($\theta$)^56^. The co-occurrence matrix depends on two variables: the relative distance, which is the number of pixels that separate two pixels, expressed in pixel numbers (d), and the orientation of those pixels ($\theta$) that specifies the direction in which the co-occurrence statistics are computed in relation to one another (theta). Common orientation angles ($\theta$) include 0$^\circ$ (horizontal), 45$^\circ$ (diagonal), 90$^\circ$ (vertical), and 135$^\circ$ (opposite diagonal). *d* = 1 and ($\theta$) = 0$^\circ$, 45$^\circ$, 90$^\circ$, 135$^\circ$ are commonly employed in calculations. In our case, we additionally defined the number of levels as 256. This parameter specifies how many grey-tone levels the GLCM takes into account. It establishes the texture analysis’s granularity. Higher levels result in more detailed texture information. The Following GLCM features were extracted in our research work:

**Contrast** reflects the intensity variation between a pixel and its neighbor across the image. It is defined by the squared difference in intensity levels of each pixel pair, emphasizing the presence of edges and other significant spatial variations in texture. This measure increases with the intensity difference, highlighting areas of high frequency and texture. The equation is derived considering the squared difference to accentuate larger disparities more significantly, where *G*(*i*, *j*) denotes the frequency of occurrence of pixel pairs with intensities *i* and *j* and can be obtained using:

7 $$\begin{aligned} \text{ Contrast } =\sum _i \sum _j|i-j|^2 G(i, j) \end{aligned}$$

**Dissimilarity** while similar to contrast, applies linear weighting to the intensity differences of pixel pairs. It assesses texture variations by calculating the absolute differences in gray levels, providing insights into the spatial distribution and heterogeneity of textures. The formula simplifies the comparison by linearly quantifying the disparity, making it sensitive to finer texture variations. It can be given by:

8 $$\begin{aligned} \text{ Dissimilarity } =\sum _i \sum _j|i-j| G(i, j) \end{aligned}$$

**Homogeneity** or the inverse difference moment (IDM), measures the smoothness of texture by assigning higher weights to pixel pairs close to each other in intensity. It prefers uniform textures by favoring values near the GLCM diagonal, thus indicating areas of consistency. It utilizes an inverse function to diminish the effect of large intensity differences, thereby prioritizing similarity and smoothness in the texture as denoted by:

9 $$\begin{aligned} \text{ Homogeneity } =\sum _i \sum _j \frac{1}{1+|i-j|^2} G(i, j) \end{aligned}$$

**Angular Second Moment (ASM)** quantifies the uniformity of gray level distribution across an image. High ASM values suggest a concentrated occurrence of certain pixel intensity pairs, indicating a more uniform texture. Conversely, low ASM values denote a broad spread of pixel pair intensities. The calculation employs squaring to emphasize the prevalence of repeated pixel pair values, thus measuring texture uniformity. It is calculated using:

10 $$\begin{aligned} \text{ ASM } = \sum _i\sum _j G(i, j)^2 \end{aligned}$$

**Energy** closely related to ASM, represents the sum of squared elements in the GLCM. This metric serves to identify regions of consistent intensity, with higher values indicating more homogeneous textures. It is essentially the square root of ASM, reflecting the texture’s uniformity through:

11 $$\begin{aligned} \text{ Energy } = \sqrt{\sum _i\sum _j G(i, j)^2} \end{aligned}$$

**Correlation** assesses the linear dependency between the gray levels of pixel pairs over the entire image. High correlation values indicate a predictable relationship, suggesting a smooth transition across pixel values. It is derived to measure how deviations from the mean correlate between pixel pairs and is given by:

12 $$\begin{aligned} \text{ Correlation } =\sum _{i} \sum _{j} \frac{(i-\mu _{i})(j-\mu _{j}) G(i, j)}{\sigma _{i} \sigma _{j}} \end{aligned}$$

where *G*(*i*, *j*) is the GLCM of an image and $\mu _i$, $\mu _j$ and $\sigma _i$, $\sigma _j$ are the mean and standard deviations of *G*(*i*, *j*).

#### Local binary pattern features

Local Binary Pattern (LBP) is a highly effective texture descriptor used in both computer vision and image analysis. Initially introduced by Ojala et al.^57^ in 1994 for texture classification, LBP serves as a local feature descriptor that encodes local texture information via the comparison of intensity values of pixels within a circular neighborhood around a central pixel (*x*, *y*), enabling the detection of local patterns between neighboring pixels. The fundamental concept lies in the substitution of intensity values for each pixel in the neighborhood with a binary code, either 0 or 1, indicating whether the intensity surpasses or falls below that of the central pixel.

The binary code is obtained by concatenating the signs of the differences between the central pixel and its neighbors. The binary codes are then used to construct a histogram of frequency occurrences, which is used as a feature vector for the image. The LBP algorithm used takes two parameters as input along with the image: radius (R) and number of neighbors (P). We defined the neighbor pixels as *P* = 8 and *R* =1.0. The LBP code of a pixel (*x*, *y*) is expressed as in Eq. (13).

13 $$\begin{aligned}{} & {} {\text {LBP}}_{P, R}=\sum _{p=0}^{P-1} S\left( g_p-g_c\right) 2^p \end{aligned}$$

14 $$\begin{aligned}{} & {} S(x)=\left\{ \begin{array}{cc} 1 &{} \text{ if } x \ge 0 \\ 0 &{} \text{ otherwise } \end{array}\right. \end{aligned}$$

where the grey value of the central pixel (*x*, *y*) is represented by $g_c$ and the intensities of the eight adjacent pixels are also denoted by $g_p$. *S* is a scaling coefficient.

### Sea-horse optimizer

This section delineates a new technique for selecting features, which is a novel nature-inspired metaheuristic algorithm that goes by the name of Sea-horse Optimizer (SHO). Taking inspiration from various types of movement, the probabilistic hunting process, and the distinct mating characteristics of seahorses that are crucial to their survival in the wild, the SHO swarm intelligence algorithm was developed to tackle complex optimization problems. The meta-heuristic approach of the SHO algorithm is characterized by a balanced trade-off between exploration and exploitation, which emulates the movement and predation behaviors of the seahorse. However, the final phase of matting is carried out when both components have terminated. Seahorses are a type of marine fish in the Syngnathidae family^58^. They are distinguished by their enlarged snouts, and prehensile tails, and also for their unusual reproductive behavior, with males carrying and incubating the eggs until they hatch.

SHO mimics the different ways in which seahorses move by using two different movement modes: Brownian motion and Lévy flight. A seahorse can either float in a spiral motion caused by the activity of sea vortices or it can drift with the waves. The predatory approach of the algorithm models the probability of the seahorse’s success or failure in capturing prey, taking into account that it has a chance of over 90% of seizing food. Furthermore, the SHO algorithm’s final phase facilitates the production of offspring while preserving vital information regarding the male parent, which promotes population heterogeneity owing to the unique trait of male pregnancy. Algorithm 1 displays the basic SHO algorithm’s pseudo-code.

#### Initialization

The SHO algorithm’s implementation begins with an initial population, which is achieved by randomly generating a set of solutions in the search space [0, 1], as given in Eq. (15). This population functions as the foundation for subsequent iterations and space exploration.

15 $$\begin{aligned} \text{ Seahorses } =\left[ \begin{array}{ccc} x_1^1 &{} \cdots &{} x_1^{\text{ Dim } } \\ \vdots &{} \ddots &{} \vdots \\ x_{P}^1 &{} \cdots &{} x_{\text{ P } }^{\text{ Dim } } \end{array}\right] \end{aligned}$$

where *Dim* signifies the dimension of the variable and *P* represents the population size.

The best individual that displays the optimum fitness level (smallest fitness value) within a minimum optimization problem is referred to as $X_{\text{ elite }}$ and is expressed as follows:

16 $$\begin{aligned} X_{\text{ elite } }={\text {argmin}}\left( f\left( X_i\right) \right) \end{aligned}$$

where *f*(.) is the value of the fitness function.

#### Movement (Exploration phase)

For the movement behavior, there are two strategies: The spiral motion, which is evidently influenced by the action of sea vortices, or the Brownian motion, affected by the action of sea waves and drifting. In the first case, the Lévy flight serves as an effective model for capturing the spiral movement of the seahorse, and it can be expressed as follows:

17 $$\begin{aligned} X_{\text{ n1 }}(t+1) =X_i(t)+{\text {Levy}}(\lambda )\left( \left( X_{\text{ elite } }(t)-X_i(t)\right) \times x \times y \times z+X_{\text{ elite } }(t)\right) \end{aligned}$$

where *x*, *y*, and *z* denote the horizontal, vertical, and depth dimensions of the spiral path, respectively. Together, these variables simulate the complex, natural searching patterns through space. The function ${\text {Levy}}(z)$ prescribes a step size based on the Lévy flight distribution, as delineated in Eq. (23,24). Here, $\rho$ represents the algal stem or leaf length with respect to logarithmic spiral constants *u* and *v* (which have constant values of 0.05). A random number $\lambda$ is generated within the range of 0 to 2, while *w* and *k* are also randomly generated within the range of 0 to 1. Additionally, a fixed value of 0.01 is assigned to the constant *s*.

18 $$\begin{aligned}{} & {} \theta ={\text {rand}} \times 2 \pi \end{aligned}$$

19 $$\begin{aligned}{} & {} x=\rho \times \cos (\theta ) \end{aligned}$$

20 $$\begin{aligned}{} & {} y=\rho \times \sin (\theta ) \end{aligned}$$

21 $$\begin{aligned}{} & {} z=\rho \times \theta \end{aligned}$$

22 $$\begin{aligned}{} & {} \rho =\mu \times e^{\theta v} \end{aligned}$$

23 $$\begin{aligned}{} & {} {\text {Levy}}(\lambda )=s \times \frac{w \times \sigma }{|K|^{\frac{1}{\lambda }}} \end{aligned}$$

24 $$\begin{aligned}{} & {} \sigma =\left( \frac{\Gamma (1+\lambda ) \times \sin \left( \frac{\pi \lambda }{2}\right) }{\Gamma \left( \frac{1+\lambda }{2}\right) \times \lambda \times 2^{\left( \frac{\lambda -1}{2}\right) }}\right) \end{aligned}$$

Secondly, to enhance the seahorse’s exploration capabilities within the search space, an additional motion strategy, namely Brownian motion, is utilized. This involves imitating the movement pattern of another seahorse that is influenced by drifting action and contributes to determining the new position of the seahorse during iteration *t*. Eq. (25) can be used to represent this scenario mathematically.

25 $$\begin{aligned} X_{\text {n1}}(t+1) = X_i(t) + {\text {rand}} \times l \times \beta _t \times \left( X_i(t) - \beta _t \times X_{\text {elite}}\right) \end{aligned}$$

where the constant coefficient, denoted by *l*, is set to 0.05. The random walk coefficient of Brownian motion is represented by $\beta$ as in Eq. (26).

26 $$\begin{aligned} \beta _t=\frac{1}{\sqrt{2 \pi }} \exp \left( -\frac{x^2}{2}\right) \end{aligned}$$

Consequently, the new position of the seahorse at iteration *t* can be formulated as presented in Eq. (27), leveraging the distinct characteristics of these two motion paradigms to enhance exploration capabilities within the search space. This integrated approach encapsulates the seahorse’s movement behaviors, embodying both the intricate spiral trajectories influenced by sea vortices and the unpredictable patterns driven by sea wave actions and drifting.

27 $$\begin{aligned} \begin{aligned} X_{\text{ n1 }}(t+1) ={\left\{ \begin{array}{ll} X_i(t)+{\text {Levy}}(\lambda )\left( \left( X_{\text{ elite } }(t)-X_i(t)\right) \times x \times y \times z+X_{\text{ elite } }(t)\right) , &{} \text{ if } r_1>0 \\ X_i(t)+ \text{ rand } \times l \times \beta _t \times \left( X_i(t)-\beta _t \times X_{\text {elite}}\right) , &{} \text {otherwise} \end{array}\right. } \end{aligned} \end{aligned}$$

where the variable $r_1$ represents a normally distributed random number that is also utilized in the process.

#### Predation (Exploitation phase)

Seahorses have two outcomes when preying on zooplankton and small crustaceans: success and failure. A random number $r_2$ with a threshold value of 0.1 is utilized to discriminate the two possibilities with a success rate of more than 90%. If $r_2$ > 0.1, the seahorse has successfully captured its prey by sneaking up and moving faster than it. If $r_2$ < 0.1, the seahorse then engages in an exploratory search of the surrounding space. The mathematical representation of this hunting pattern is expressed by Eq. (29).

28 $$\begin{aligned}{} & {} \alpha =\left( 1-\frac{t}{T}\right) ^{\frac{2 t}{T}} \end{aligned}$$

29 $$\begin{aligned}{} & {} \begin{aligned} X_{\text{ n2 }}(t+1) ={\left\{ \begin{array}{ll} \alpha \times \left( \left( X_{\text{ elite } }(t)- \text{ rand } \times X_{\text{ n1 }}(t)\right) +(1-\alpha ) \times X_{\text{ elite } }(t)\right) , &{} \text{ if } r_2>0 \\ (1-\alpha ) \times \left( \left( X_{\text{ n1 }}(t)- \text{ rand } \times X_{\text{ elite } }(t)\right) +(\alpha ) \times X_{\text{ n1 }}(t)\right) , &{} \text {otherwise} \end{array}\right. } \end{aligned} \end{aligned}$$

where $X_{\text{ n1 }}(t)$ is the seahorse’s updated position following movement at iteration *t*, $\alpha$ is a directly decreasing parameter with each iteration that modifies the seahorse’s moving step size when chasing prey as stated in Eq. (28), and $r_2$ is a random value between zero and one. The total iteration number is denoted by *T*.

#### Reproduction

The SHO algorithm sorts the population into males and females according to their fitness values, with the best half of each gender serving as fathers and mothers, respectively. This ensures the inheritance of desirable traits and prevents the clustering of new solutions. The algorithm then randomly mates males and females to produce offspring. To simplify the process, each pair is assumed to only have one child. The mathematical representation for the chosen seahorses for reproduction, as well as the resultant offspring, is outlined in Eqs. (30) and (31), respectively.

30 $$\begin{aligned}{} & {} \begin{aligned}{}&\text{ fath } =X_{\text{ n2 } }^{\text{ s }}(1: \lceil P / 2\rceil ) \\&\text{ moth } =X_{\text{ n2 } }^{\text{ s }}({ \lceil P / 2\rceil } / 2+1: \lceil P / 2\rceil ) \end{aligned} \end{aligned}$$

31 $$\begin{aligned}{} & {} X_i^{\text{ offs } }=r_3 X_i^{\text{ f } }+\left( 1-r_3\right) X_i^{\text{ m } } \end{aligned}$$

where $X_{\text{ n2 } }^{\text{ s }}$ represents all the sorted $X_{\text{ n2 }}(t)$ in ascending order according to their fitness, and *fath* and *moth* are the male and female populations, respectively. $r_3$ is a random number between [0, 1], and *i* denotes a positive integer that falls within the interval $(1, \lceil P / 2\rceil )$. $X_i^{\text{ f } }$ and $X_i^{\text{ m } }$ correspond to randomly selected members originating from the respective female and male populations.

**Algorithm 1:**
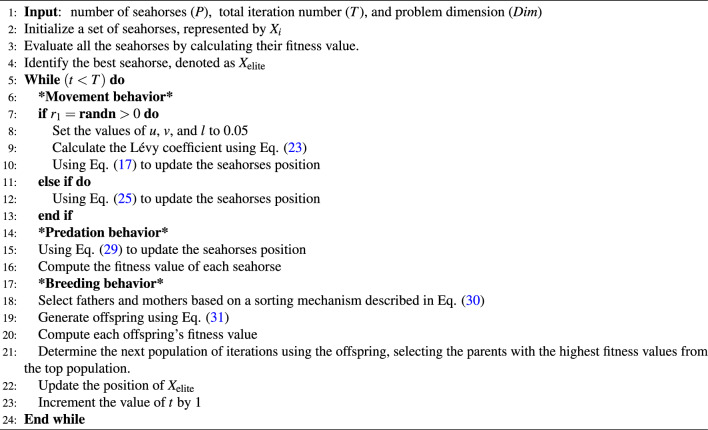
Steps of the original SHO.

### Sine cosine algorithm

In 2016, Mirjalili et al.^34^ introduced the SCA algorithm, a meta-heuristic optimization technique that has been proven to be a successful approach for solving a wide array of optimization problems, including FS. The algorithm is inspired by the sine and cosine functions, and one of its advantages is that it is relatively simple and easy to implement, while still being effective for many optimization problems^35^. For FS tasks, it has been demonstrated to outperform other MAs, such as PSO and GA.

The SCA algorithm begins by generating a number of initial search agents and then repetitively iterates to identify the best solution. Each search agent represents a possible solution, and its position in the search space corresponds to a specific set of features. The algorithm modifies the position of each agent, utilizing equations grounded on the sine and cosine functions, along with a set of parameters that govern the exploration and exploitation of the search space. Algorithm 2 describes the pseudocode for the SCA methodology. The objective function is evaluated for each search agent, with the agents updated in accordance with Eq. (32):

32 $$\begin{aligned} X_{\text{ i }}(t+1)= {\left\{ \begin{array}{ll}X_i(t)+b_1 \times \sin \left( b_2\right) \times \left| b_3 X_{\text{ elite } }(t)-X_i(t)\right| , &{} b_4<0.5 \\ X_i(t)+b_1 \times \cos \left( b_2\right) \times \left| b_3 X_{\text{ elite } }(t)-X_i(t)\right| , &{} \text {otherwise}\end{array}\right. } \end{aligned}$$

where $X_i(t)$ is the current solution’s position in the iteration *t*, $b_1$, $b_2$, and $b_3$ are generated randomly between 0 and 1. $X_{\text{ elite } }(t)$ indicates the best search agent position in dimension *i*. $b_4$ is generated randomly in [0, 1].

The dimensions of sine and cosine in Eq. (32) are adjusted using the following equation to strike a harmonious equilibrium between the exploration and exploitation stages. Here, *t* represents the present iteration, *T* represents the overall iteration count, and $a=2$ represents a constant value.

33 $$\begin{aligned} b_1=a-t \frac{a}{T} \end{aligned}$$

**Algorithm 2:**
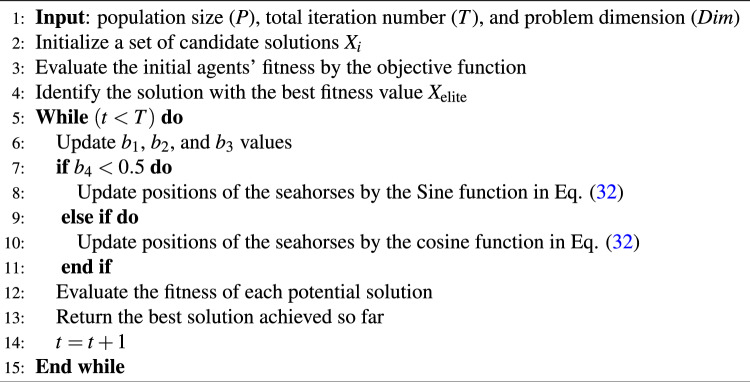
The SCA Pseudo code algorithm

## The proposed method for MS detection

**Figure 1:**
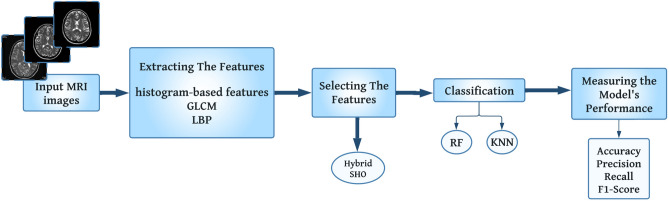
The structure of the proposed MS detection method.

The developed system is intended to aid radiologists in the classification of MR images showing MS lesions in the brain. As depicted in Fig. 1, our methodology comprises three main phases, and the training dataset includes 80% of the images, while the remaining 20% is allocated to the testing dataset. Initially, the images undergo the process of feature extraction, during which a feature vector consisting of an extensive suite of different texture features is derived using the texture feature analysis methods discussed in the above sections. The next phase involves the process of FS through the utilization of the modified SHO approach, which is employed to identify a subset of significant features from the set of input features. In the third phase, different classifiers (KNN, Random Forest (RF)) are used to recognize unhealthy from healthy brain images. Each classifier’s performance has been evaluated based on a number of different variables.

**Figure 2:**
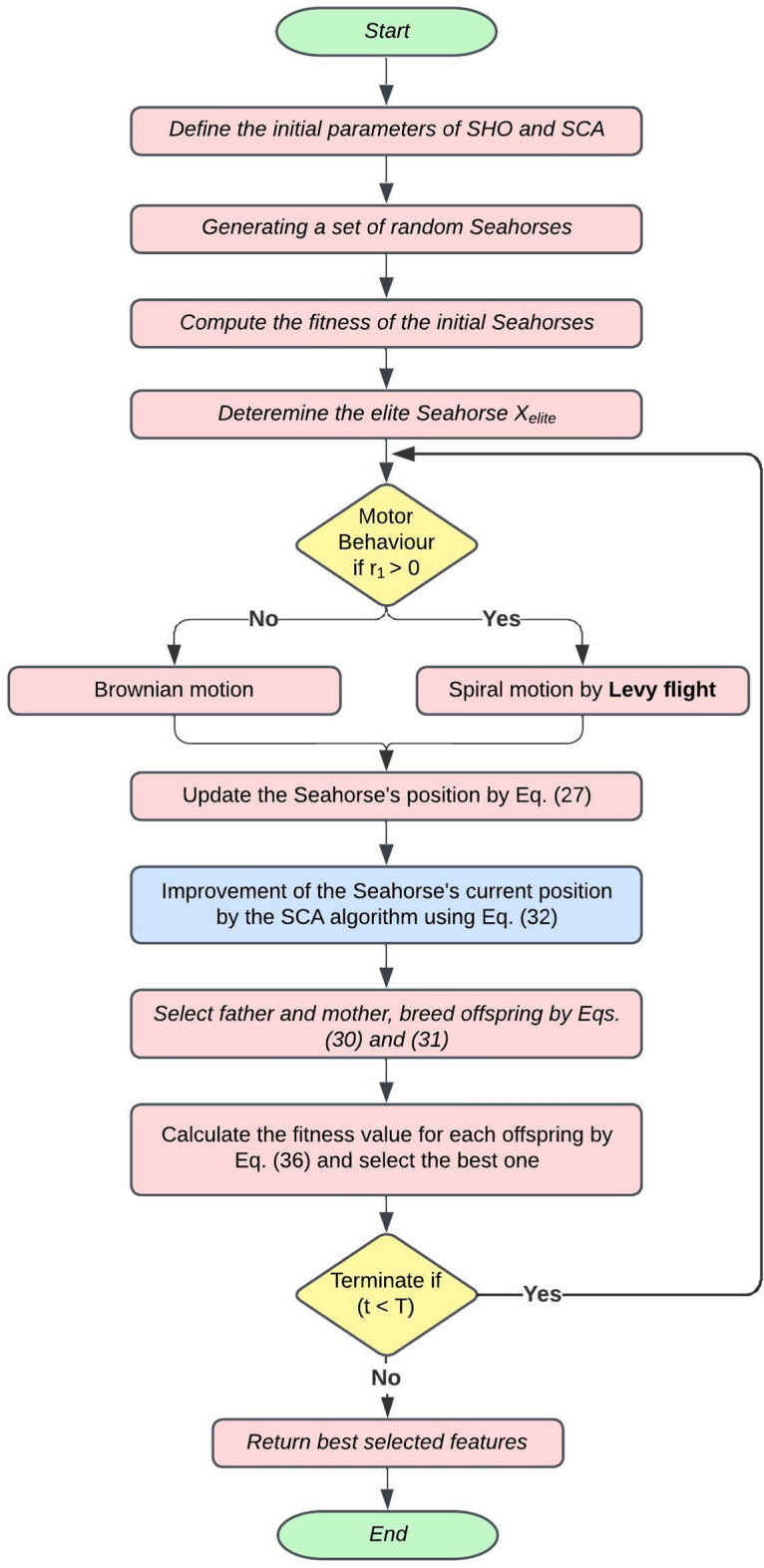
Flowchart of the enhanced SHOSCA algorithm.

In general, the main structure of the proposed Sea-horse Optimizer Sine Cosine Algorithm (SHOSCA) as an FS model is shown in Fig. 2, where the first step is to define the parameters and initialize a set random solution, and the search agents attempt to identify near-optimal solutions inside the search space [0, 1]. KNN, a machine learning algorithm, has been used for training instances and evaluating the efficacy of the selected feature set due to its excellent performance in classification. Once the random initialization is complete, a fitness function is used to assess the quality of the initial group of seahorses. The resulting population, determined through fitness assessment, undergoes motion, foraging, and breeding mechanisms.

The SHOSCA algorithm employs a dual-phase movement strategy for optimizing search solutions, incorporating unique biological and mathematical concepts to enhance its effectiveness. Initially, the algorithm simulates a spiral motion, reminiscent of seahorses navigating through vortices, directed towards the most promising solution, denoted as $X_{\text {elite}}$. This phase utilizes Lévy flights to dynamically adjust the spiral rotation angle, effectively minimizing excessive local exploitation and expanding the search area around current local optima. In contrast, the second phase adopts Brownian motion to model seahorse movement influenced by sea waves, facilitating a comprehensive exploration of the search space. After this, the algorithm updates the seahorses’ positions employing the trigonometric functions integral to the SCA, using either a sine or cosine function to steer the search towards or away from the target solution, based on a probabilistic threshold.

Subsequently, the seahorses are next exposed to mating behavior, where the resulting population is split into male and female groups according to their respective fitness values. Half of the population serves as fathers, while the remaining half serves as mothers. This division is pivotal for the algorithm as it mimics the unique reproductive behavior of real-world seahorses, where males carry the offspring, thus ensuring the selection of superior traits for the next generation and mitigating the potential for solution over-localization. Through random mating, each pair produces a single offspring, simplifying SHOSCA’s implementation while emphasizing genetic diversity in offspring generation, bolstering the algorithm’s ability to amalgamate genetic features for the discovery of novel solutions. Once the termination condition is satisfied, the algorithm will automatically terminate.

The following provides a more in-depth explanation of the developed hybrid algorithm implementation. The Movement behavior, signifying the exploration phase in the foundational SHO algorithm, updates the search agents’ positions relative to the solution’s position, as delineated by Eqs. (18,19,20,21,22)-(27). The SCA, executed after the movement behavior and detailed in Eq. (32), enhances the exploitation phase. Following the update mechanism, the seahorses are subjected to the mating behavior, as governed by Eqs. (30) and (31). Algorithm 3 illustrates the operation of the SHOSCA approach.

### Feature selection by SHOSCA

The developed SHOSCA, a FS algorithm, is described. The steps of SHOSCA are given in detail as follows:

The SHOSCA algorithm begins by defining the initial SHO and SCA settings, after which the SHO generates *N* random candidate solutions as represented by Eq. (34).

34 $$\begin{aligned} X_{i, d}=l b_d+{\text {rand}}\times \left( u b_d-l b_d\right) \end{aligned}$$

where $X_{i, d}$ is the dimension *d* of the $i^{t h}$ solution; $u b_j$ and $l b_j$ are the lower and upper bounds of dimension *d*, respectively; *rand* produces random values uniformly between 0 and 1.

Before calculating the fitness function, each solution $X_{i}$ should be expressed as a binary string based on a random threshold value between [0, 1]. The elements that correspond to ones will be selected, and those that correspond to zeros will be ignored (irrelevant features) using the equation below. Where $X_{i, d}^{bin}$ is the updated binary position of dimension *d* of a candidate solution $X_i$.

35 $$\begin{aligned} X_{i, d}^{bin}= {\left\{ \begin{array}{ll}1 &{} \text{ if } X_i>0.5 \\ 0 &{} \text{ otherwise } \end{array}\right. } \end{aligned}$$

In addition, the fitness function is evaluated using Eq. (36) to assess the quality of the specified features defined by the search agents. Both the classification error rate and the selected number of features constitute the fitness function.

36 $$\begin{aligned} Fitness=\alpha \times E_{X_i}(t)+(1-\beta )\left( \frac{\left| S\right| }{|D|}\right) , \alpha =0.99, \beta =1-\alpha \end{aligned}$$

where $E_{X_i}(t)$ is the error of the classification made by the learning algorithm (the KNN classifier, in our case), *S* is the number of selected features, *D* is the total number of features in the original dataset, and $\alpha$ and $\beta$ are constants that are used to find a balance between the error $E_{X_i}(t)$ and the number of features *S* chosen in the search space [0, 1] by the proposed method, SHOSCA.

The population that emerges following the assessment of fitness is subsequently exposed to movement, foraging, and mating behaviors of sea horses in nature, enabling the evolution of fresh solutions. Predation is substituted in our novel hybrid technique by the SCA algorithm’s position update mechanism, an intermediate algorithm used to improve the population’s worst outcomes.

The best solution, $X_{\text{ elite }}$, is chosen when the resulting possible solutions are once again assessed using the objective function, see Eq. (36). The aforementioned steps are repeated in a cyclic manner until the condition for termination is satisfied, which is characterized by the attainment of the upper limit of iterations that are permitted.

The SHOSCA-retrieved subset of features is employed to decrease the dataset’s dimensionality. For the purpose of performance evaluation, the dataset is partitioned randomly into two distinctive sections, with 80% designated for training, while the remaining 20% is set for testing. For classifying results, the KNN algorithm is used and it should be noted that the process is executed 25 times.

**Algorithm 3:**
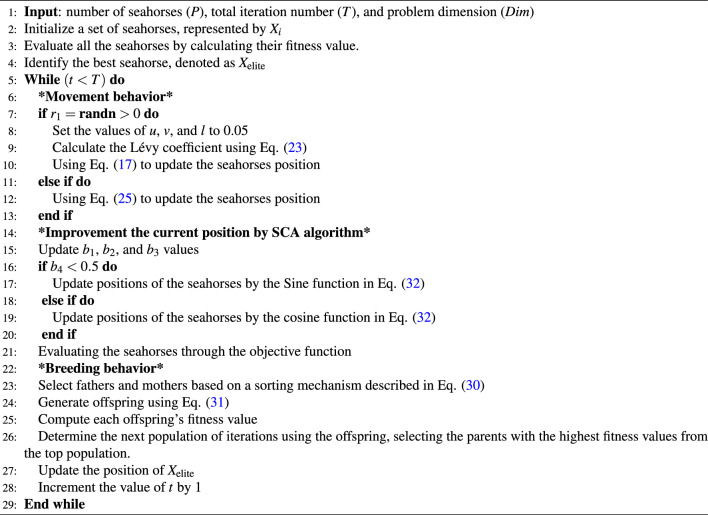
Steps of the proposed MS detection approach.

## Experimental results and discussion

In this section, we present the outcomes of our proposed MS identification model. The work was performed on a Dell laptop running Windows 10 operating system, with a 2.40 GHz i5-1135G7 CPU and 8GB RAM, using Python 3.10 programming language.

Our objective is to evaluate the effectiveness of the developed algorithm and its optimization performance. To achieve this, we conducted two main test cases. First, we conducted our method on the Genuine-Rad-MS dataset (GRMS dataset), a collection of retrospective brain MRI scans. Results were compared with the original SHO as well as the state-of-the-art optimization methods. In addition to performing a sensitivity analysis to confirm the efficacy of our methodology, we executed a comparison between the KNN and RF classifiers to determine which classifier more effectively leverages the selected features for MS identification. Furthermore, a comparative assessment between T2-Weighted and Fluid-Attenuated Inversion Recovery (FLAIR) images was undertaken, aimed at substantiating the model’s versatility and its utility as a supportive tool for clinicians in the early diagnosis of MS. Subsequently, we tested the algorithm on the open-access eHealth laboratory dataset comprising only 38 MS patients. The results of our novel approach were compared with those of previous studies performed using this public dataset. Two classifiers, RF and KNN, were used to assess how well the developed technique performed when choosing features.

### Performance metrics:

The SHOSCA algorithm, a hybrid FS method, is run 25 times with an overall iteration count of 50, and the initial population size is set to 10. Configuration parameters for the SHOSCA algorithm are listed in Table 1. To determine the most valuable feature subsets, the relevant subsets are assessed using various evaluation metrics, including classification accuracy, mean and best fitness, worst fitness, standard deviation, average selected size, execution time, and the Friedman test. Here is a rundown of what will be considered during the evaluation:

**Table 1:** Configuration settings of the SHOSCA algorithm and other selected MAs, highlighting essential parameters such as the number of initial solutions, total iterations, code runs, and specific values for the k-value in KNN and other algorithmic constants.

Parameter	Value
Number of initial solutions	10
Total iteration (T)	50
Number of code runs	25
k-value in KNN	5
Threshold value	0.5
Constant coefficient (l) of SHO	0.05
logarithmic spiral constants (u & v) of SHO	0.05
Levy component ($\beta$) of SHO	1.5
Parameter of SCA (a)	2
Parameter of WOA (b)	1

**Mean accuracy**: Since each algorithm is executed 25 times with random initialization, it is essential to acquire information regarding the mean accuracy (Mean Acc) as specified by:

37 $$\begin{aligned} \text{ Mean } \text{ Acc } =\frac{1}{R} \frac{1}{M} \sum _{r=1}^R \sum _{j=1}^{M}\left( c_j==t_j\right) \end{aligned}$$

where *R* denotes the count of runs performed on the algorithm, *M* is the number of test samples, $c_i$ represents the label outputted by the classifier for a specific point *i*, and $t_i$ represents the class label assigned to that particular data point *i*.

**Mean fitness:** The mean fitness measures how well a set of solutions performs on average throughout multiple runs of an optimization technique, as represented by:

38 $$\begin{aligned} \text{ Mean } \text{ fitness } =\frac{1}{R} \sum _{i=1}^R F i t_i^* \end{aligned}$$

where *R* denotes the total count of runs and $F i t_i^*$ is the optimal solution for *ith* run.

**Best fitness:** Referring to the objective function of a metaheuristic optimizer, the best fitness can be defined as the lowest value attained when running an optimization algorithm *M* times. This notion is quantified using:

39 $$\begin{aligned} \text{ Best } _{f i t}={\text {Min}}_{i=1 }^R F i t_i^* \end{aligned}$$

where *R* is the number of times the optimization process has been run and $F i t_i^*$ signifies the most optimal outcome obtained from a specific run denoted by *i*.

**Worst fitness:** It indicates the maximum solution reached among the best solutions when an optimization process is run *R* times, as given by:

40 $$\begin{aligned} \text{ Worst } _{f i t}={\text {Max}}_{i=1 }^R F i t_i^* \end{aligned}$$

where *R* reflects how many times the optimization method was run to narrow down the features to be picked out and $F i t_i^*$ is the best solution derived from the *ith* execution.

**Mean feature selection size:** It indicates the average size of the selected features as determined by an optimization approach, in comparison to the overall number of features set forth by:

41 $$\begin{aligned} \text{ Average } \text{ selection } \text{ size } =\frac{1}{R} \sum _{i=1}^R \frac{{\text {size}}\left( F i t_i^*\right) }{D} \end{aligned}$$

where size(x) is the number of values for the vector *x*, *R* is the number of code runs, $F i t_i^*$ signifies the optimum solution generated at the *ith* execution, and *D* is the number of features present in the initial dataset.

**Standard Deviation (Std):** It is an indication of how much variability there is in the algorithm’s performance across multiple executions, as expressed by:

42 $$\begin{aligned} std=\sqrt{\frac{1}{R-1} \sum _{i=1}^{R}\left( F i t_i^*-\text {Mean fitness}\right) ^2} \end{aligned}$$

where *R* is the number of runs, $F i t_i^*$ is the best solution resulting from the *ith* run, and mean fitness is the average defined in Eq. (38).

**Mean Execution time:** The average duration, in milliseconds, that an optimization algorithm necessitates to identify the most suitable subset of features from a specified dataset, as outlined in:

43 $$\begin{aligned} \text{ Mean } \text{ time } =\frac{1}{R} \sum _{i=1}^R T_r \end{aligned}$$

where $T_r$ is the amount of time an optimization method takes on the *ith* run and *R* is the number of times the code is run.

**Friedman test:** It is a non-parametric statistical technique used to examine whether differences between several groups or treatments are statistically significant. It is frequently employed in experimental contexts when data from numerous trials or experiments are gathered. In the context of MAs for FS, the Friedman test can be used to compare the performance of different optimization algorithms on different FS problems.

### First case: Performance of developed method using genuine-rad-MS dataset

Here, we will discuss the outcomes of the FS process carried out by the hybrid SHOSCA algorithm based on the GRMS dataset. Our analysis was conducted using different approaches. Initially, we compared the performance of our hybrid approach with the original SHO. In addition, we compared the effectiveness of SHOSCA with three well-established population-based MAs: namely, Sine Cosine Algorithm (SCA), Whale Optimization Algorithm (WOA), and Harris Hawk Optimization (HHO).

#### Dataset description

In this retrospective study, MRI scans of 425 individuals were collected, comprising 163 healthy individuals (with an average age of 32.35 ± 10.30 years) and 262 patients clinically diagnosed with MS at various disease stages (with an average age of 31.26 ± 10.34 years). The study adhered to the principles outlined in the Declaration of Helsinki and received approval from the Ethics Committee at the Faculty of Science, Mansoura University (Code number: Sci-phy-M-2022-122, issued on October 3, 2022). Data were gathered anonymously in this analysis as the images were stored in DICOM format. Informed consent from patients was waived by the Institutional Review Board (IRB) of Mansoura Faculty of Medicine at Mansoura University due to the retrospective nature of the study. Comprehensive details regarding the patients’ demographics are provided in Table 2. To reduce computation time in the preprocessing step, the images were converted into JPG file format with a size of 512 $\times$ 512 pixels and a resolution of 24 dpi for each patient slice. Individuals with general contraindications to MRI (e.g., metal implants, cochlear implants, or other ferromagnetic materials), those with a history of neurological or psychiatric conditions, those with neoplastic lesions, those taking psychotropic medications, or those who have any other condition that could compromise their safety during the scanning procedure, were not considered in collecting the dataset for the study. Aiming to expose our model to a wide variety of disease manifestations and thereby enable it to learn as broad a range of clinical scenarios as possible, including severely affected patients and newly diagnosed individuals with a low number of lesions, we applied no exclusion criteria based on the duration, burden, or activity of the disease.

**Figure 3:**
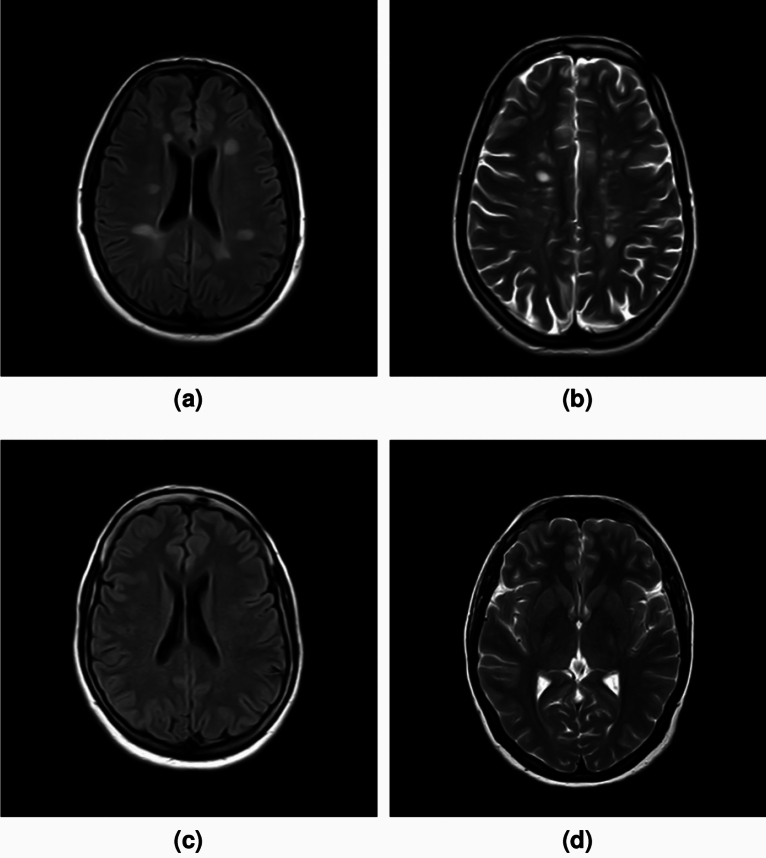
Brain slices samples from the GRMS dataset. (**a**) & (**b**) Two MS slices with multiple plaques. (**c**) & (**d**) Two healthy brain slices.

MRI scans were conducted using a 1.5 T Philips Ingenia scanner. Our proposed methodology was applied to the T2-weighted images (TR = 5280 msec; TE = 110 msec; 24 contiguous 5 mm slices) and the FLAIR images (TR = 11000 msec; TE = 130 msec; and TI = 2800 msec; 24 contiguous 5 mm slices), to enhance our analysis. However, for each of the 425 patients’ brain MRIs, 4 images were specifically excluded-those from both the T2 and FLAIR sequences that included sections from the vertex and the base of the skull, as these images did not contain brain regions. Following this refinement, the remaining images were processed and analyzed by our algorithm. A couple of brain slices from the GRMS dataset, showcasing both T2 and FLAIR images, are illustrated in Fig. 3.

**Table 2:** Demographic Characteristics for the GRMS Dataset, Detailing Gender Distribution, Average Age with Standard Deviation, Age Range, and Total Number of MRI Scans for MS Patients and HCs.

Class	Male	Female	Total	Male Age	Female Age	Age (min - max)
MS	63	199	262	34.31 ± 10.63	30.31 ± 10.09	15–65
Normal	54	109	163	28.35 ± 10.05	34.28 ± 9.90	13–62

#### Comparison to other FS algorithms

Results produced by the developed methodology, based on the T2-weighted images from the GRMS dataset, are compared with those obtained by the fundamental SHO algorithm and other FS algorithms to determine the efficacy of the proposed MS detection method (SHOSCA). Table 3 summarizes the results, from which we can observe the following points. First, analysis of the findings demonstrates that SHOSCA is superior to the original SHO, WOA, HHO, and SCA in terms of the following four key evaluation metrics. Accuracy (Acc), which indicates the proportion of right predictions made by the model (both true positives and true negatives) in relation to the total number of predictions, achieved 91.2471%. The Precision (Prec), which quantifies the proportion of true positive predictions (correctly predicted positive observations to the total predicted positives), was 90.7619%. Recall (Rec), also known as the sensitivity or true-positive rate, indicates the proportion of actual positives that were identified correctly; this scored 86.3030%. The F1 score, a balanced harmonic mean of precision and recall providing a more comprehensive view of model performance when dealing with imbalanced classes, was recorded at 88.4382%.

Regarding fitness, the proposed approach’s objective function values, encompassing mean, standard deviation, best fitness, and worst fitness, were compared to the algorithms mentioned earlier. According to the results presented in Table 3, it is evident that SHOSCA performs better than the other algorithms, but there is no major difference between it and the SCA in terms of performance since it offers virtually the same results. The hybrid SHOSCA algorithm has an average fitness (Avr fit) of 0.1099 and the lowest standard deviation (Std fit) of 0.0105. It also has the best fitness (Best fit) of 0.0944 and the worst fitness (Worst fit) of 0.1295. This indicates that the algorithm delivers the most consistent and superior performance overall.

Additionally, by considering the average size of the chosen features, it can be deduced that SHOSCA surpasses HHO and WOA algorithms in picking out relevant subsets of features. Furthermore, the SCA algorithm has a lower count of selected features compared to the hybrid SHOSCA. Conversely, the SHO algorithm in its original form proves to be the most effective in eliminating irrelevant features. Our proposed approach shows a tendency to select a comparatively larger set of features, thereby providing a more extensive exploration of the feature space.

In the context of computational time, surpassing the others, the WOA showcases the fastest CPU time, averaging at 1.7474 seconds. However, in contrast, SCA ranked as the second most effective in this aspect. As for the proposed SHOSCA method, it had the longest computation time among the evaluated algorithms, requiring an average of 8 seconds. This longer duration can be attributed to the complexity of the algorithm, which involves a combination of three different mechanisms to search for optimal feature subsets.

**Table 3:** Performance comparison between proposed SHOSCA and other optimization algorithms, highlighting its excellence in metrics such as accuracy, precision, recall, and F1 score for MS detection. It evidences SHOSCA’s enhanced efficiency in FS and model optimization, despite a longer CPU time, underscoring its potential as a robust tool in clinical diagnostics.

	Feat Size	Acc	Prec	Rec	F1 Score	CPU Time	Avr fit	Std fit	Best fit	Worst fit
SHO	**9.48**	85.7882	83.3532	79.7576	81.3509	3.9979	0.1213	0.0149	0.0933	0.1514
HHO	98.92	88.6118	86.2826	84.1212	85.1439	4.0070	0.1257	0.0238	**0.0699**	0.1556
WOA	76	89.4118	88.0679	84.3636	86.0967	**1.7474**	0.1270	0.0237	0.0815	0.1544
SCA	46.48	89.9294	89.3919	84.1212	86.6184	3.1534	0.1124	0.0145	0.0825	0.1401
Proposed SHOSCA	63.76	**91.2471**	**90.7619**	**86.3030**	**88.4382**	8.0064	**0.1099**	**0.0105**	0.0944	**0.1295**

*Significant are in bold.

**Figure 4:**
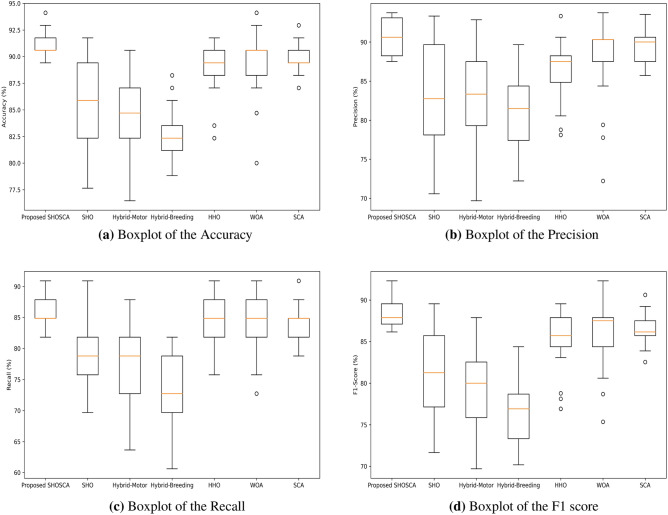
Boxplot of proposed SHOSCA versus state-of-the-art optimization approaches. (**a**) Accuracy; (**b**) Precision; (**c**) Recall; (**d**) F1 Score.

To showcase the reliability of the developed SHOSCA when compared to the original SHO and other MAs, Fig. 4 illustrates boxplots for Acc, Prec, Rec, and F1 score. These graphs provide details on the 25th percentile, 75th percentile, minimum, maximum, and median values of the measured metrics. The median is represented by the central line, not the mean, and outliers are individually marked with a ’°’ symbol. It is quite apparent from the boxplots that neither plain SHO nor any of the other algorithms could outmatch the proposed MS detection method in terms of Acc, Prec, Rec, and F1 score. These boxplots authenticate the preeminence of the developed technique, as evidenced by the impressive values accomplished by RF concerning the selected features.

**Figure 5:**
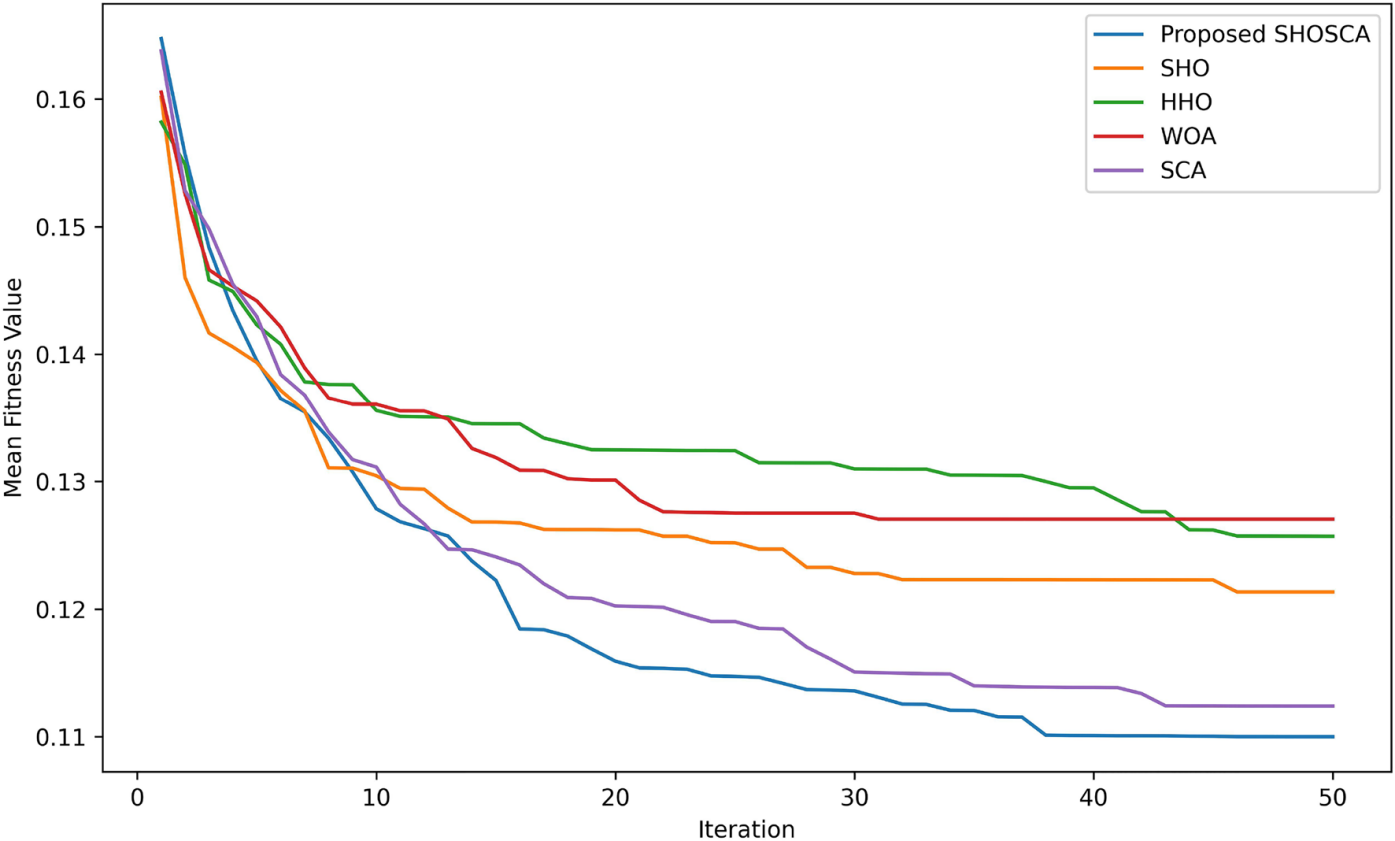
Convergence curve of the proposed method versus other optimization algorithms.

Moreover, the convergence curve of the proposed SHOSCA, in comparison to the basic SHO and other algorithms, is presented in Fig. 5. The convergence curves illustrated in the figure are established based on the correlation between the average best fitness values and the number of iterations, with a maximum of 50 iterations. As shown, the proposed SHOSCA algorithm exhibits the highest convergence accuracy among the other algorithms, converging faster than the other four algorithms.

Assessing the effectiveness of the proposed approach in detecting MS involved performing a statistical analysis using the Friedman test. This test was used to compare the performance of SHOSCA with other MAs employed in this study. Its purpose was to identify significant distinctions among various sets of observed data. The resulting p-values and rankings, which provide valuable insights into these differences, have been documented in Table 4. The mean rank represents the average ranking of each algorithm across different performance metrics. The p-value reveals how likely it is that the differences between the algorithms are indeed statistically significant.

The findings presented in Table 4 demonstrate that the SHOSCA approach exhibits competitiveness when compared to alternative methods, as evidenced by its attainment of the highest rank in terms of Acc, Prec, Rec, and F1 score. On the other hand, Hybrid Motor and Hybrid Breeding have the worst rank. Regarding the mean fitness value, our proposed method ranked first, followed by the SCA and SHO algorithm. This ranking suggests that the “SHOSCA” algorithm achieves the best fitness among the evaluated algorithms. It’s important to note that the SCA algorithm follows closely behind in the rankings. In terms of CPU time, our proposed method had the highest rank among them all while the WOA algorithm achieved the lowest rank. This is probably because it may involve more complex computations or additional steps in its execution. As for the selected feature size, our method ranks last, while the basic SHO ranks first, followed by the hybrid breeding algorithm.

However, to determine if these mean rank differences are statistically significant, we can look at the corresponding p-values. A smaller p-value indicates a higher level of statistical significance. In this case, all the p-values are extremely small, ranging from 3.03E-26 to 2.17E-02, indicating highly significant differences among the algorithms for all performance metrics.

These findings lead us to the conclusion that the performance of the algorithms varies significantly across all measures examined. Generally speaking, the developed SHOSCA technique outperforms the other comparable methods in terms of identifying the most relevant characteristics.

**Table 4:** Friedman test results for the MS detection method (Hybrid SHOSCA) versus other optimization approaches based on performance metrics like feature size, accuracy, precision, recall, F1 score, CPU time, and average fitness. SHOSCA consistently ranks highest in performance metrics, highlighting its effectiveness in diagnosis despite a longer CPU time. The statistical significance of these outcomes, as shown by low p-values, underscores SHOSCA’s superior performance in enhancing MS detection accuracy.

	Proposed SHOSCA	SHO	Hybrid Motor	Hybrid breeding	HHO	WOA	SCA	*P*-value
Feat Size	5.84	2.38	2.54	**2.22**	5.12	4.9	5	1.11E-14
Accuracy	**6.06**	3.1	2.68	1.8	4.46	5	4.9	2.22E-14
Precision	**6.2**	3	2.7	2.02	3.82	5.04	5.22	1.90E-14
Recall	**5.64**	3.36	2.7	1.7	4.78	5.08	4.74	1.87E-13
F1 score	**5.92**	3.16	2.54	1.94	4.66	4.92	4.86	4.93E-13
CPU Time	7	4.36	3.24	5.84	4.2	**1**	2.36	3.03E-26
Avr fit	**2.88**	3.92	4.06	3.94	4.96	4.64	3.6	2.17E-02

*Significant are in bold.

### Sensitivity analysis for the proposed MS detection method

In this section, we compared our approach with other hybrid models of the original Seahorse Optimizer to evaluate its quality, specifically using T2-weighted images from the GRMS dataset. We applied two experiments over the basic SHO also using the sine cosine algorithm with the same maximum number of iterations and population sizes. In the first trial, referred to as **Hybrid SHOSCA (Motor)**, we introduced a new approach to enhance the exploration capabilities of the SHO by incorporating the SCA. To achieve this, we substituted the SHO’s movement behavior equation, as denoted by Eq. (27) with Eq. (32), which corresponds to the updating position mechanisms of the SCA. Then, in the second trial, referred to as **Hybrid SHOSCA (Breeding)**, the SHO’s mating behavior was replaced by the SCA algorithm. This was done by replacing Eqs. (30) and (31) with the trigonometric functions of the SCA using Eq. (32).

The outcomes of the two trials, in comparison to the developed SHOSCA algorithm which employs the RF classifier, are displayed in Table 5. Based on these metrics, the Proposed Hybrid SHOSCA algorithm consistently outperforms the other two algorithms, “Hybrid SHOSCA (Motor)” and “Hybrid SHOSCA (Breeding),” in terms of Acc, Prec, Rec, and F1 Score. Across all these metrics, it achieves the highest values, demonstrating superior overall performance in terms of classification accuracy, precision in identifying positive instances, recall in capturing true positive instances, and F1 Score as a balanced measure between precision and recall.

In terms of fitness, The Proposed Hybrid SHOSCA algorithm consistently had the lowest average fitness value of 0.1099, indicating better fitness compared to the other algorithms. It also had the lowest standard deviation (0.0105), suggesting more stability in its performance. The best fitness of 0.0944 indicates that it achieved the best fitness value among the algorithms, while the worst fitness of 0.1295 indicates that the algorithm performs well overall but may encounter challenges in certain cases. Our proposed method selects the highest number of features, with 63.76 features, followed by the “Hybrid SHOSCA (Motor)” algorithm with 9.52 features, and the “Hybrid SHOSCA (Breeding)” algorithm with 4.36 features. As for the execution time, the Proposed Hybrid SHOSCA algorithm had the longest CPU run time of 8 seconds, followed by the “Hybrid SHOSCA (Breeding)” algorithm with 4.9187 seconds and the “Hybrid SHOSCA (Motor)” algorithm with 3.5809 seconds. The “Hybrid SHOSCA (Motor)” algorithm had the shortest CPU run time.

**Table 5:** Comparison of the proposed SHOSCA algorithm with other hybrid Sea-horse Optimizer variations, emphasizing superiority in accuracy, precision, recall, and F1 score for MS detection. Despite a longer processing time, SHOSCA shows optimal performance in fitness metrics, underlining its effectiveness and reliability over other hybrids in enhancing diagnostic precision.

	Feat size	Acc	Prec	Rec	F1 Score	CPU Time	Avr fit	Std fit	Best fit	Worst fit
Hybrid SHOSCA (Motor)	9.52	84.6588	82.9226	76.6061	79.4621	**3.5809**	0.1237	0.0213	**0.0817**	0.1636
Hybrid SHOSCA (Breeding)	**4.36**	82.7294	80.8739	73.2121	76.6171	4.9187	0.1212	0.0106	0.0933	0.1399
Proposed SHOSCA	63.76	**91.2471**	**90.7619**	**86.3030**	**88.4382**	8.0064	**0.1099**	**0.0105**	0.0944	**0.1295**

*Significant are in bold.

**Figure 6:**
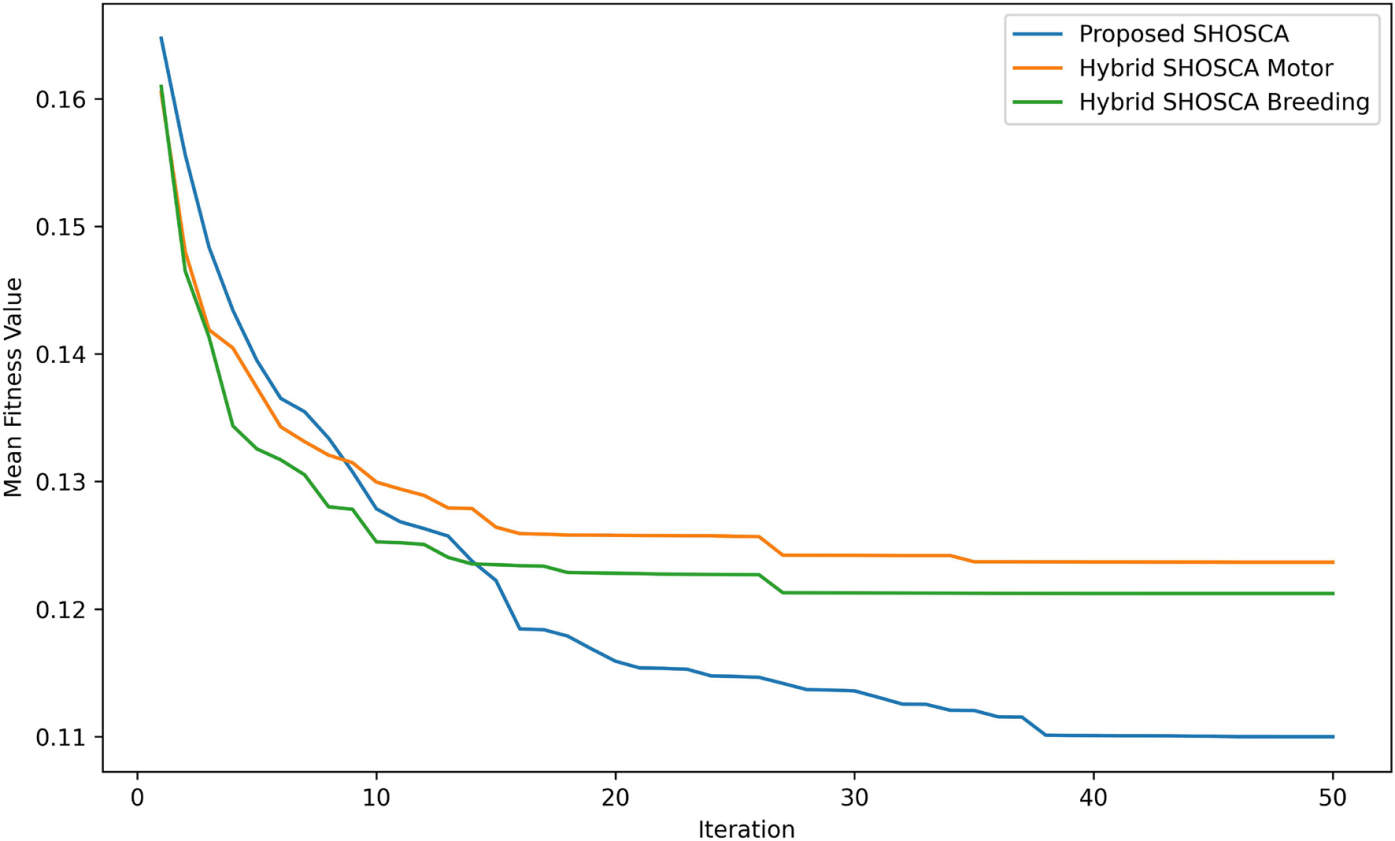
Convergence curve of the developed method in comparison to other hybrid models of the SHO.

Figure 6 displays the convergence curve for SHOSCA and the other two hybrid models derived from the original SHO algorithm. The figure indicates that the SHOSCA method we developed exhibits exceptional results compared to the other two hybrid methods, in terms of both convergence speed and efficiency. This is evidenced by the fact that the other models require a longer period of time to reach the optimal solution. These results provide further support for the effectiveness of our MS detection method, which effectively balances the exploration and exploitation phases. Additionally, our hybrid approach, which combines SHO and SCA, elevates the FS capabilities of the original SHO, leading to a higher-quality selection of the most important features.

In summary, the Proposed Hybrid SHOSCA algorithm demonstrates superior performance in terms of accuracy, F1 score, and FS compared to the other two algorithms. However, it requires more execution time compared to Hybrid SHOSCA (Motor) and Hybrid SHOSCA (Breeding). The proposed algorithm also exhibits a lower standard deviation and better fitness values, indicating its stability and effectiveness.

### Comparison between classifiers for the proposed hybrid SHOSCA

The following experiment was carried out to evaluate the effectiveness of the SHOSCA algorithm, established as the top-performing FS design when paired with other classification techniques, specifically the K-nearest neighbors method. In the case of KNN, we utilized a k parameter of 5 to test the GRMS dataset, whereas in the RF method, we tested the dataset using n_estimators of 100. The aim was to categorize the T2 MRI image dataset into MS and normal classes. The chosen features from our suggested FS method served as the input data for the KNN and RF classifiers.

**Figure 7:**
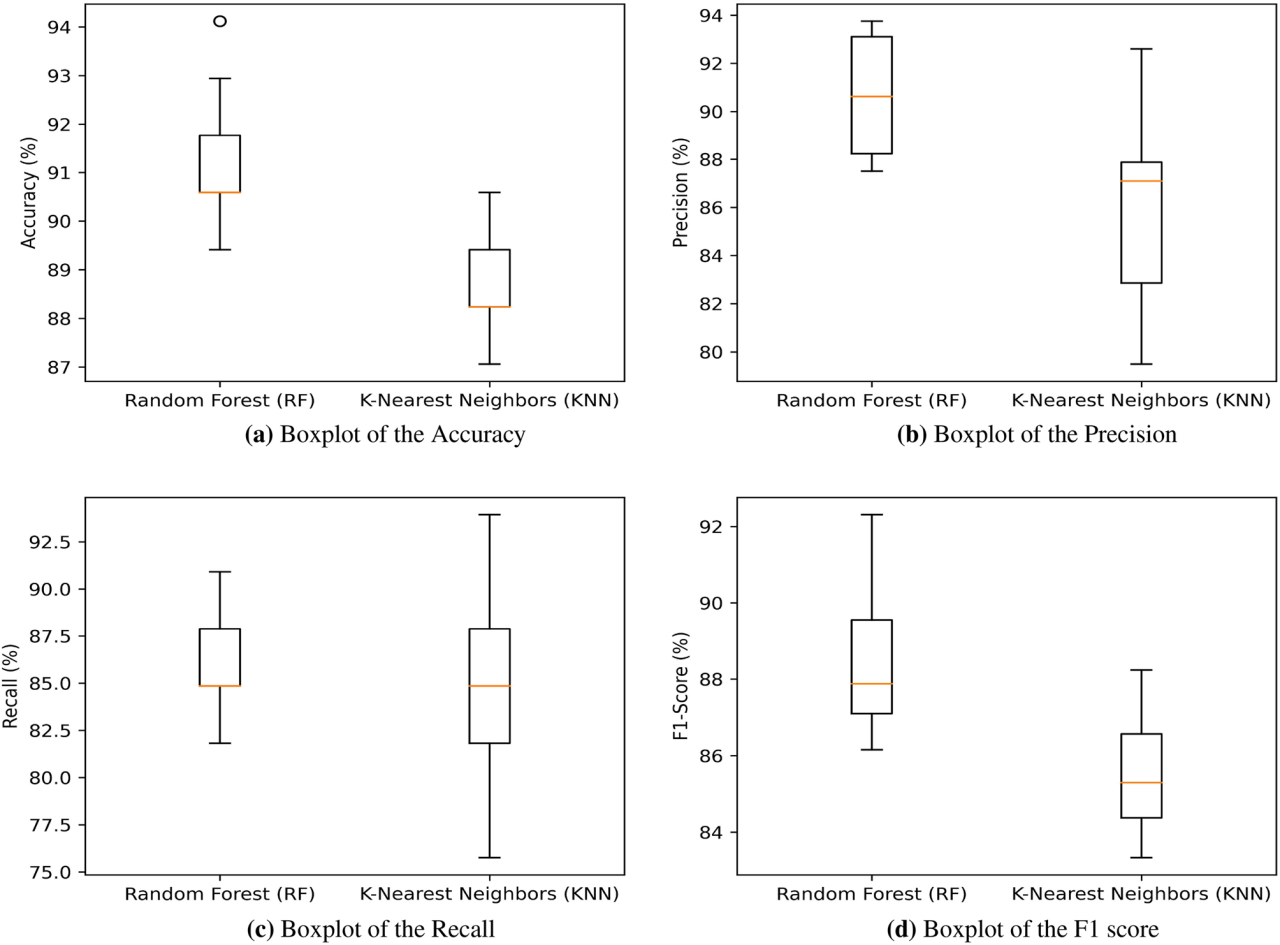
Boxplots for comparison between Random Forest (RF) and K-Nearest Neighbors (KNN) classifiers for the MS detection method. (**a**) Accuracy; (**b**) Precision; (**c**) Recall; (**d**) F1 Score.

The results derived from both the KNN and RF classifiers are recorded in Table 6. A variety of assessment criteria were utilized to assess their individual effectiveness with regards to Acc, Prec, Rec, and F1 score. The RF classifier yielded better results, boasting an Acc of 91.2471%, Prec of 90.7619%, Rec of 86.3030%, and an F1 score of 88.4382%. These values reflect a high volume of true positive outcomes (as highlighted by the high precision), a substantial capability to identify positive instances (evidenced by the recall), and a generally balanced performance (as depicted by the F1 score). Even though the KNN classifier displayed slightly diminished results, it nonetheless exhibited solid performance with an accuracy of 88.8%, precision of 85.9797%, recall of 85.3333%, and an F1 score of 85.5194%. This confirms the effective operation of the proposed Hybrid SHOSCA FS method across various classifier types, even though it seems particularly well-suited to tree-based approaches like Random Forests, as visualized by the boxplots in Fig. 7.

**Table 6:** Comparison between RF and KNN classifiers for the proposed method based on the GRMS dataset, showing the RF classifier excels in accuracy, precision, recall, and F1 score. This highlights RF’s effectiveness in MS detection when paired with Hybrid SHOSCA.

Proposed hybrid SHOSCA	Acc	Prec	Rec	F1 score
KNN	88.8	85.9797	85.3333	85.5194
RF	**91.2471**	**90.7619**	**86.3030**	**88.4382**

*Significant are in bold.

### Comparative analysis of T2-weighted and FLAIR image-based algorithms

The continuous evolution of AI-based diagnostic models for MS necessitates the exploration of various imaging modalities to enhance diagnostic precision. While T2-weighted MRI scans have been the cornerstone for initial model training, their high sensitivity to white matter anomalies can lead to nonspecific results. Incorporating FLAIR imaging, which suppresses the signal from cerebrospinal fluid, provides a clearer differentiation of MS lesions, potentially reducing false positives and improving the clinical applicability of the AI model.

The comparative analysis, as detailed in Table 7, between the models trained on T2 and FLAIR images yielded notable results. The FLAIR-based model, employing the RF classifier, demonstrated a higher accuracy (92.94% vs. 91.25%), precision (95.67% vs. 90.76%), and F1 score (90.03% vs. 88.44%), with a modest trade-off in recall (85.12% vs. 86.30%) and an increase in CPU processing time (12.36 vs. 8.01 seconds). Notably, the feature size in the FLAIR-based model expanded from 63.76 to 91.16, reflecting a broader and more detailed extraction of characteristics pertinent to MS detection from the MRI scans. Additionally, the fitness metrics, while exhibiting a slight uptick in average and standard deviation, suggest a reliable performance of the algorithm across different evaluations. The consistency of the model is further underscored by the stability of the best and worst fitness scores when transitioning from T2-weighted to FLAIR imaging. This stability is essential for ensuring the model’s reliability in a clinical setting, where varying patient presentations demand consistent algorithm performance. These results suggest that FLAIR images can enhance the model’s ability to accurately identify MS, with a marginal increase in computational demand.

**Table 7:** Comparative Analysis of Diagnostic Performance Metrics between T2-Weighted and FLAIR MRI Image-Based AI Models for MS Detection, highlighting that the FLAIR model achieves higher accuracy, precision, and F1 score, albeit with a larger feature size and longer CPU time, utilizing the RF classifier.

	Feat size	Acc	Prec	Rec	F1 score	CPU time	Avr fit	Std fit	Best fit	Worst fit
T2	**63.76**	91.2471	90.7619	86.303	88.4382	**8.0064**	**0.1099**	**0.0105**	0.0944	**0.1295**
FLAIR	91.16	**92.94118**	**95.66871**	85.125	**90.0345**	12.35661	0.1181	0.0109	**0.0943**	0.1413

*Significant are in bold.

The inclusion of FLAIR imaging in the AI-based diagnostic model marks a significant advancement in the non-invasive detection of MS. The improved precision reflects the model’s enhanced ability to discern MS-specific lesions, reinforcing its potential as a supportive tool for clinicians in the early and accurate diagnosis of MS. Future work will focus on leveraging the complementary strengths of both T2-weighted and FLAIR imaging to further refine the model and achieve the highest possible accuracy and specificity in MS diagnosis.

### Second case: Performance of developed method using eHealth lab dataset

**Figure 8:**
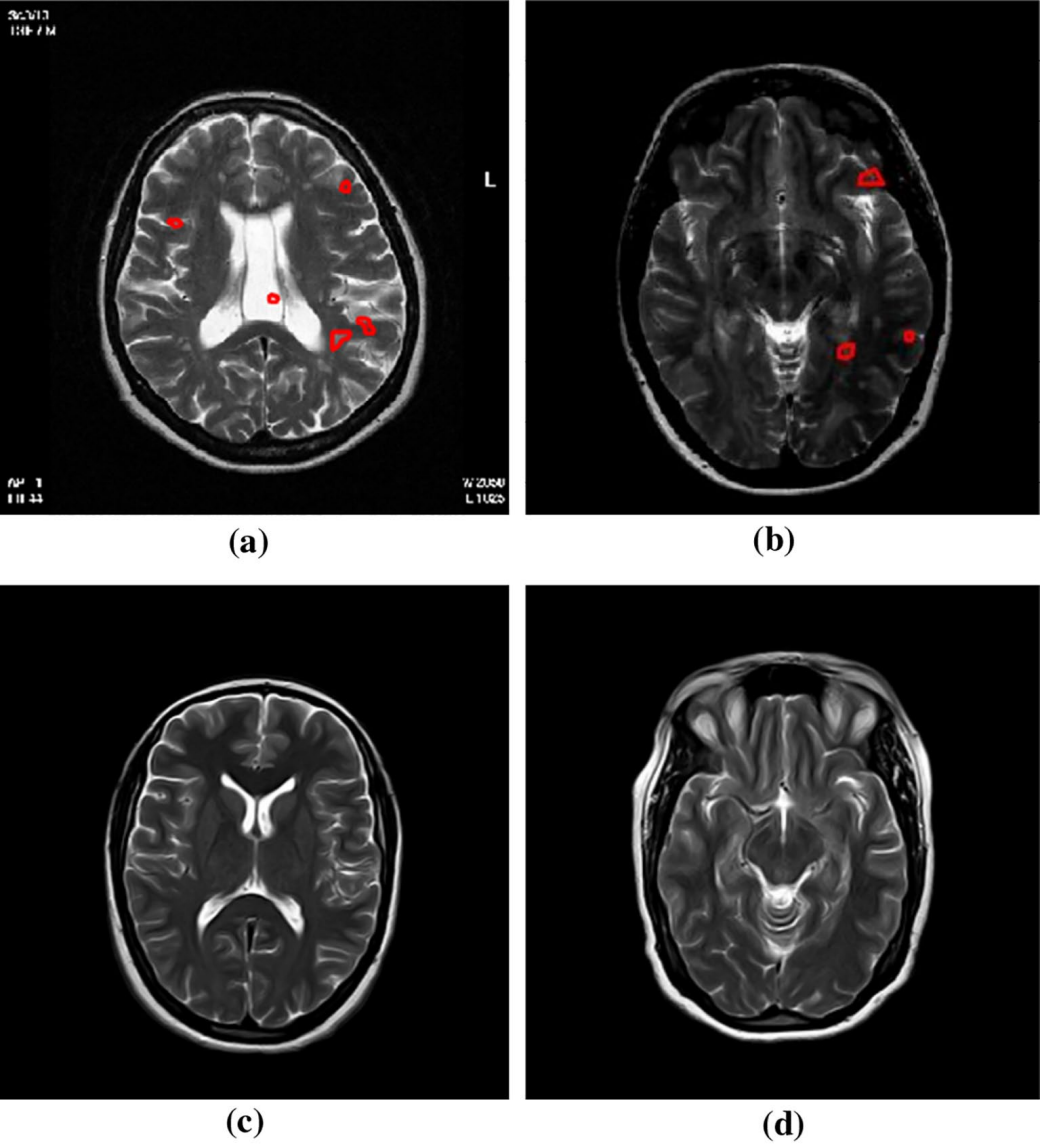
Sample of brain slices from the eHealth lab and GRMS datasets. (**a**) & (**b**) Two MS slices from the eHealth lab dataset with delineated plaques (Lesions are surrounded by red lines). (**c**) & (**d**) Two healthy brain slices from our GRMS dataset.

In this scenario, the images were derived from two independent sources. The MS images were obtained from the eHealth laboratory^59^ comprises only 38 MS patients, with an average age of 34.1 ±10.5 years, 17 of whom were male and 21 were female. Detailed information regarding the data is available in reference^45^. An expert consultant radiologist with more than 10 years of experience in MRI reporting reviewed the dataset and selected the included brain slice images. We acquired a total of 676 slices by selecting only those that were associated with plaques. For comparison with normal brain studies, we included 760 T2-weighted brain images from 38 HCs who matched the age and gender distribution of the eHealth lab data from the GRMS dataset which includes normal brain MRI scans from 163 healthy individuals. Table 8 summarizes the demographics of both MS patients and HCs. In addition, images from both datasets were resized to 512 $\times$ 512 pixels. Figure 8 provides sample images taken from both the eHealth and GRMS datasets.

**Table 8:** Demographic characteristics of two Datasets: MS patients from eHealth laboratory and healthy individuals from GRMS dataset.

Dataset	Provider	Number of subjects	Age	Gender (m/f)	Number of slice
Multiple Sclerosis	eHealth	38	34.1 ± 10.5	17/21	676
Healthy Individuals	GRMS	38	30.3 ± 9.4	14/24	760

In order to ensure comparability and facilitate analysis of brain images, the Interscan Normalization technique is employed to address the variations in image intensities caused by different scanning machines. This process, known as inter-scan normalization, involves matching the intensity ranges of the dataset’s sources. One effective method employed for this purpose is histogram stretching (HS)^60^, which enhances the dynamic range of the original brain images. By applying the HS equation to each pixel’s coordinates, the normalized image, denoted as *O*, is obtained from the original brain image, *N*, by applying the HS equation as described below in Eq. (44), which adjusts the pixel intensities based on their coordinates (*x*, *y*) and the minimum ($N_{\min }$) and maximum ($N_{\max }$) intensity values.

44 $$\begin{aligned} O(x, y)=\frac{N(x, y)-N_{\min }}{N_{\max }-N_{\min }} \end{aligned}$$

In this experiment, we compared our proposed technique with other MS identification methods from machine and deep learning studies, such as Haar Wavelet Transform, principal component analysis, and Logistic Regression (HWT + PCA + LR)^61^, Wavelet Entropy and Feedforward Neural Network Trained by Adaptive Genetic Algorithm (WE + FNN + AGA)^44^, Hu Moment Invariant and Feedforward Neural Network Trained by Particle Swarm Optimization (HMI + FNN + PSO)^46^, gray level co-occurrence matrix and Feedforward Neural Network (GLCM + FNN)^45^, multiscale amplitude-modulation frequency-modulation (MAMFM)^62^, gray level co-occurrence matrix and ensemble Learning along with LogitBoost algorithm (GLCM + ensemble + LogitBoost)^41^, gray level co-occurrence matrix and the gray level run length matrix (GLCM + GLRLM)^63^, 6-layer stochastic pooling convolutional neural network (6l-CNN)^64^, discrete wavelet transform, principal component analysis, and least squares support vector machine (DWT + PCA + LS-SVM)^65^, Wavelet Entropy and Hybridization of Biogeography-Based Optimization and Particle Swarm Optimization (WE + HBP)^66^, discrete wavelet transform and probabilistic principal component analysis with random forests (DWT + PPCA + RF)^67^, fractional Fourier entropy, multilayer perceptron, and Self-adaptive Three-segment-encoded Jaya algorithm (FRFE + MLP + ST-Jaya)^16^, Biorthogonal Wavelet Transform, RBF Kernel Principal Component Analysis, and Logistic Regression (BWT + RKPCA + LR)^38^, Minkowski-Bouligand dimension, single hidden layer neural network, and three-segment representation biogeography-based optimization (MBD + SHLNN + TSR-BBO)^68^, stationary wavelet entropy and k-nearest neighbors (SWE + KNN)^37^, and biorthogonal wavelet features and fitness-scaled adaptive genetic algorithm (BWF + FAGA)^69^. All the techniques were tested on the same open-access dataset as ours. The comparative analysis is listed in Table 9.

From the results in Table 9, it is evident that the algorithm that performed the poorest was HWT + PCA + LR, with an accuracy value of lower than 90%. All of the remaining algorithms outperformed it by more than 90%. Our Hybrid SHOSCA approach, when employing both the KNN and RF classifiers on the eHealth dataset, demonstrates exceptional levels of accuracy and sensitivity. The KNN classifier reached slightly superior values of 97.97% accuracy and 98.89% sensitivity, while the Random Forest algorithm yielded a close 97.77% accuracy and 97.73% sensitivity. This slight but critical disparity in performance between the two algorithms suggests that the KNN classifier performs slightly better on the eHealth dataset when combined with our handcrafted feature extraction and a well-suited FS algorithm, outperforming other state-of-the-art approaches. In conclusion, our findings strongly suggest that our approach offers significantly superior performance, highlighting the potential of this combined methodology.

**Table 9:** Comparison of the Proposed approach with RF and KNN classifiers outperforms the state-of-the-art MS detection methods conducted on the eHealth lab dataset, achieving top accuracy and sensitivity. Specifically, the combination with KNN reaches the highest marks, showcasing the effectiveness of this method in MS detection.

MS detection methods	Accuracy	Sensitivity
HWT + PCA + LR^61^	89.72	-
WE + FNN + AGA^44^	91.95	91.91
HMI + FNN + PSO^46^	91.70	91.67
GLCM + FNN^45^	92.75	92.75
MAMFM^62^	93.83	94.08
GLCM + ensemble + LogitBoost^41^	94.91	95.79
GLCM + GLRLM^63^	95.14	95.27
6l-CNN^64^	95.82	95.98
DWT + PCA + LS-SVM^65^	96.21	95.86
WE + HBP^66^	96.72	96.15
DWT + PPCA + RF^67^	96.40	96.01
FRFE + MLP + ST-Jaya^16^	97.39	97.40
BWT + RKPCA + LR^38^	97.76	97.12
MBD + SHLNN + TSR-BBO^68^	97.80	97.78
SWE + KNN^37^	97.94	96.15
BWF + FAGA^69^	97.89	98.00
GLCM + LBP + Hybrid SHOSCA + RF (ours)	**97.77**	**97.73**
GLCM + LBP + Hybrid SHOSCA + KNN (ours)	**97.97**	**98.89**

*Significant are in bold.

## Conclusions and future work

In this research, an innovative approach has been established for the identification of multiple sclerosis (MS) utilizing a combination of texture feature analysis, histogram-based features, gray level co-occurrence matrix (GLCM), and local binary pattern (LBP) techniques. The method incorporates the Hybrid SHOSCA algorithm for selecting the most relevant features. The algorithm’s distinctive quality is its hybridization with the SCA method, which enhances the exploitation capabilities of the Sea-horse Optimizer, making it particularly potent for high-dimensional datasets. The effectiveness of the algorithm in reducing the dimensionality of the MRI datasets, without compromising the crucial information necessary for accurate diagnoses, is a notable finding. This feature is instrumental in improving the computational efficiency of the overall process, making it more suitable for real-world applications. The analysis of our experimental results derived from the two different datasets demonstrates the superiority of our approach (Texture features + Hybrid SHOSCA + RF) over existing state-of-the-art methods, achieving higher accuracy and performance in identifying MS lesions in MRI images. The success of this method supports its potential application in assisting healthcare professionals to more accurately and swiftly diagnose MS, enhancing patient prognosis through earlier intervention and treatment planning.

Moving forward, we present several future research directions including acquiring more data to validate the algorithm, testing advanced classifiers, applying the method to other imaging modalities, and investigating additional feature extraction and deep learning techniques to enhance detection accuracy. Furthermore, a vital extension of our work involves applying our algorithm across a broader spectrum of neurological diseases, broadening its clinical applicability. By continuing to innovate in algorithm development and pursuing these research directions, we aim to improve the accuracy and early detection of MS, thus benefiting patients by enabling timely and effective treatment.

## Data Availability

The raw data presented in this article can be shared upon reasonable request by contacting the corresponding author (Mohamed G. Khattap).
